# Excavation of resources of *Streptomyces* species in frozen soils of the Qinghai-Tibet Plateau based on RpfA protein of *Streptomyces coelicolor*

**DOI:** 10.3389/fmicb.2025.1557511

**Published:** 2025-04-08

**Authors:** Yuxiao Xie, Jingjing Liu, Jun Ma, Nan Shi, Xiumin Zhang

**Affiliations:** ^1^College of Life Sciences, Hebei University, Baoding, China; ^2^Key Laboratory of Microbial Diversity Research and Application of Hebei Province, Baoding, China; ^3^Engineering Research Center of Microbial Breeding and Conservation, Baoding, China

**Keywords:** *Streptomyces coelicolor*, resuscitation-promoting factor, RpfA protein, frozen soils of the Qinghai-Tibet Plateau, resources of *Streptomyces* species

## Abstract

This study is aimed at the actual demand for exploring new species resources of *Streptomyces*, and aims to solve the technical bottleneck of *Streptomyces* isolation and culture. A new method was established based on the resuscitation function of the RpfA protein from *Streptomyces coelicolor* CGMCC 4.1658^T^ to isolate unculturable or difficult-to-culture *Streptomyces* species, and it was applied to explore *Streptomyces* species resources in special habitats in the frozen soils of the Qinghai-Tibet Plateau. The RpfA protein of *S. coelicolor* was heterologously expressed and validated for its *in vitro* activity. The purified RpfA protein was then used to isolate *Streptomyces* from soil samples in the frozen soils of the Qinghai-Tibet Plateau, followed by an investigation into the impact of the RpfA protein on the cultivability of *Streptomyces* species. The results showed that the RpfA protein had a significant promoting effect on the germination of spores of both *S. coelicolor* itself and other species of the *Streptomyces* genus, and when a suitable concentration of RpfA protein was added to the culture medium, it could significantly improve the culturability of members of phylum *Actinomycetota*, especially *Streptomyces* species. In addition, many new species of the genus *Streptomyces* and other genera of phylum *Actinomycetota* were discovered. This study provides a new approach for further exploring *Streptomyces* species resources in special environments such as the Qinghai-Tibet Plateau and developing new biologically active substances produced by *Streptomyces*.

## 1 Introduction

*Streptomyces* are known for producing a wide variety of antibiotics and are considered to be an endless treasure trove of natural product biosynthesis diversity. Approximately two-thirds of the antibiotics used clinically are produced by *Streptomyces* (Barka et al., 2016; Bérdy, 2012; Gao et al., 2018; Olanrewaju and Babalola, 2019). However, in recent decades, a large number of *Streptomyces* species have been repeatedly isolated, leading to a decreasing chance of discovering new antibiotics. Moreover, most *Streptomycetes* cannot be cultured under laboratory conditions because they are in a state of being “uncultivable” or “not yet cultivated,” and thus have not been discovered so far, and they are the source of many unusual bioactive natural products (Lackner et al., 2017; van Dorst et al., 2016). Research has shown that the production of antibiotics by *Streptomyces* is species-specific (Liu et al., 2013), therefore, exploring new *Streptomyces* species resources is an effective way to solve the difficulty of discovering antibiotics. For the *Streptomyces* isolation and cultivation technology, past research has usually designed suitable culture media for *Streptomyces* growth (Kuester and Williams, 1964; Stewart, 2012; Suutari et al., 2002), but these traditional strategies limited the discovery of new species. Therefore, it is urgent to develop new isolation strategies and achieve breakthroughs in isolation and cultivation techniques to explore new resources of *Streptomyces* species.

The next-generation sequencing technology reveals that there remains an extremely high level of unknown (currently “uncultivable”) microbial diversity in nature, particularly in extreme environments (Li et al., 2021; Locey and Lennon, 2016; Pham and Kim, 2012). Meanwhile, when *Streptomyces* is confronted with adverse growth conditions, it forms dormant spores with conspicuously thicker cell wall (Cava and de Pedro, 2014; Sexton et al., 2020; Vollmer et al., 2008) and no significant metabolic activity (Kell and Young, 2000), which has led to “unculturable” or difficult-to-culture state in laboratory cultures (Lopez Marin et al., 2021). The permafrost of the Qinghai-Tibet Plateau serves as a representative of extreme environments, which it features long-term low temperatures, a severe deficiency of liquid water, and limited available nutrients, making *Streptomyces* strains within it more prone to form dormant spores with low metabolic activity. These challenges have significantly hindered the isolation of microorganisms.

Resuscitation-promoting factor (Rpf) was first discovered by Mukamolova et al. (1998) in the supernatant of *Micrococcus luteus*. This protein can stimulate the resuscitation of dormant *M. luteus* cells at picomolar concentrations (pmol/L), was named as Resuscitation-promoting factor (Rpf) (Mukamolova et al., 1999). Studies have shown that proteins in the Rpf family belong to the class of hydrolytic glycosyltransferases, similar to lysozymes and lytic transglycosylases, and are able to cleave the β-1,4-glycosidic bond between N-acetylglucosamine (GlcNAc) residues and N-acetylmuramic acid (MurNAc) residues in bacterial peptidoglycan (Maione et al., 2015). Previous researches have demonstrated that Rpfs can promote the germination and growth of dormant spores (Nikitushkin et al., 2016; Sexton et al., 2020; Sexton et al., 2015). Rpfs proteins are widely distributed among Gram-positive bacteria within the phylum *Actinomycetota*. Bioinformatics analysis has revealed that *rpf* genes in different species vary in length, genetic structure, host specificity, and activity (Flärdh and Buttner, 2009; Ravagnani et al., 2005; Tantivitayakul et al., 2020). However, all *rpf* genes encode proteins that contain a conserved domain with lysozyme-like activity (the Rpf-like domain), which is approximately 70 amino acids (Ravagnani et al., 2005). *S. coelicolor* can encode five Rpfs proteins (RpfA - RpfE), all Rpf proteins structurally possess a C-type lysozyme-like domain, and their active sites have a conserved Glu, all of which are secreted proteins, unlike the Rpfs proteins of *Mycobacterium tuberculosis* (Sexton et al., 2015). It has been 22 years since the Rpf protein in *M. luteus* was first reported to be able to stimulate the recovery of dormant bacteria. During this period, the application of Rpf has been extended from the aspects of pathogen containment (Coletta-Filho et al., 2006), vaccine development (Romano et al., 2012) and diagnostic testing (Arroyo et al., 2018) to the research field of introducing Rpf protein into polluted environment remediation and sewage biological treatment system (Su et al., 2021; Su et al., 2013). The introduction of Rpf offers a novel approach for reviving difficult-to-culture microorganisms. However, there are few systematic studies on the effect of Rpf for cultivation of soil *Streptomyces*.

The RpfA protein of *S. coelicolor* can promote the resuscitation of high G + C Gram-positive bacteria, making it possible to successfully isolate dormant *Actinomycetes* in the environment. In this experiment, the RpfA protein of *S. coelicolor* was heterologously expressed in *Escherichia coli*, and the recombinant Rpf protein was verified for *in vitro* activity and added to the isolation medium to evaluate its culture effect on soil *Streptomyces*. The aim is to explore a new method for mining *Streptomyces* strain resources. Therefore, this study established a new method for isolating unculturable or difficult-to-culture *Streptomyces* species based on the resuscitation function of the RpfA protein from *S. coelicolor*, and applied it to excavate *Streptomyces* species resources in extreme environments of frozen soils of the Qinghai-Tibet Plateau. The results showed that the RpfA protein from *S. coelicolor* has a stimulatory effect on the spore germination of both itself and other *Streptomyces* species. Furthermore, the RpfA protein can significantly improve the cultivability of members of phylum *Actinomycetota*, especially *Streptomyces* species, of frozen soils of the Qinghai-Tibet Plateau, and some potential new species were isolated. This study provides a new approach for further exploring *Streptomyces* species resources in unique environments of the Qinghai-Tibet Plateau and developing new biologically active substances derived from *Streptomyces*.

## 2 Materials and methods

### 2.1 Strains and plasmids

The experimental strains and plasmids used in this study are listed in Table 1.

**Table 1:** Strains and plasmids.

Strains/plasmids	Descriptions	Sources
Strains		
*E. coli* DH5α	Cloning and plasmid maintenance	Sangon Biotech
*E. coli* BL21 (DE3)	Protein expression	Sangon Biotech
*Streptomyces coelicolor* CGMCC 4.1658^T^	Amplification of the *rpfA* gene	China General Microbiological Culture Collection Center, CGMCC
*Streptomyces griseus* CGMCC 4.1419^T^	Verification of RpfA protein activity	China General Microbiological Culture Collection Center, CGMCC
*Streptomyces laurentii* JCM 5063^T^	Verification of RpfA protein activity	Stored in this lab
*Streptomyces niveus* JCM 4251^T^	Verification of RpfA protein activity	Stored in this lab
*Streptomyces lusitanus* HBU208066^T^	Verification of RpfA protein activity	Isolated from Baiyangdian riparian soil, Stored in this lab
Plasmids		
pMD18-T	Ampr cloning vector	TaKaRa
pMD18-T-*rpfA*	pMD18-T containing the *rpfA* gene from *S. coelicolor* strain CGMCC 4.1658^T^	This study
pET 28a(+)	Ampr expression vector	TaKaRa
pET 28a(+)-*rpfA*	pET 28a(+) containing the *rpfA* gene from strain *S. coelicolor* CGMCC 4.1658^T^	This study

### 2.2 Medium and growth conditions

*S. coelicolor* CGMCC 4.1658^T^, *S. griseus* CGMCC 4.1419^T^, *S. laurentii* JCM 5063^T^, *S. niveus* JCM 4251^T^, *S. lusitanus* HBU208066 were cultured in R_2_A medium (Reasoner and Geldreich, 1985) at 28°C; *E. coli* DH5α and *E. coli* BL21 (DE3) were cultured in LB medium at 37°C. R_2_A and Glucose-Tryptone culture media (Chen et al., 2007) were used to isolate *Actinomycetota* strains from frozen soils of the Qinghai-Tibet Plateau.

### 2.3 Preparation of recombinant RpfA protein from *Streptomyces coelicolor*

The *rpfA* gene and its encoded protein from *S. coelicolor* were subjected to multiple sequence alignment and protein domain prediction using the online software UniProt (accessed on January 29, 2024)^1^ and HMMER (accessed on January 29, 2024).^2^ Genomic DNA of *S. coelicolor* CGMCC 4.1658^T^ was extracted using the “Chelex100” method (de Lamballerie et al., 1992), and PCR amplification of *rpfA* gene was performed using the primers *rpf*-F 5′-CAGTAC **CATATG** GCCACCGCGTCCGAGTGGGAC-3′ and *rpf*-R 5′-CGAAGT **GGATCC** TTACTTCAGGTGCAGCTGCTG-3′ (restriction sites *Nde* I/*Bam*H I used for cloning purposes are indicated in bold and underlined). The purified PCR product was ligated to the *Nde* I/*Bam*H I-digested pET-28a (+) plasmid and transformed into *E. coli* BL21(DE3) competent cells. The recombinant cells were cultivated in LB medium containing kanamycin (50 μg/mL), induced with 1 mM/L IPTG, collected, and washed twice with phosphate-buffered saline (PBS). After sonication to disrupt the cells, centrifugation was performed at 4°C 10,000 rpm for 20 min, and both the supernatant and precipitate were collected, and then analyzed by SDS-PAGE, respectively. The RpfA target protein was purified using Ni-NTA-agarose column (Suzhou Biodragon Technology Co., Ltd., BDTL0011-10). The concentration of RpfA protein was determined using the Lowry method (Waterborg and Matthews, 1994). Finally, the purified recombinant RpfA protein was stored at −20°C for subsequent experiments.

### 2.4 Verification of the spore germination-promoting activity of the RpfA protein

Sixty 16S rRNA gene sequences of type strains belonging to species of the genus *Streptomyces* were retrieved from the GenBank database.^3^ Phylogenetic clustering analysis was conducted using MEGA 11.0 software. According to the formation of distinct phylogenetic groups, one species from each group was selected as a representative to assess the promotion of the RpfA protein of *S. coelicolor* on spore germination activity in its own and other *Streptomyces* species. Spore suspensions of the different species were prepared and coated on R_2_A plates. Spore germination rates were determined by adding 0.1, 1, 10% (v/v) RpfA protein in above R_2_A plates as experimental group and without adding RpfA protein as control group, respectively. In addition, in order to verify the resuscitation and stimulation function of RpfA protein, sterilized inactivated RpfA protein was used as the inactivated control group. These R_2_A plates were incubated at 28°C for 72 h. Colony counts on the R_2_A solid medium were enumerated every 12 h, and the spore germination rate was calculated as follows: spore germination rate = [number of germinated spores (CFU/mL)/total number of spores in the spore suspension (CFU/mL)] × 100%. The above experiments were repeated 3 times.

To further verify the stimulatory effect of the RpfA protein on spore germination, the BacTiter-Glo™ Microbial Cell Viability Assay Kit (BacTiter-Glo™, Catalog No. G82300, purchased from Madison, Wisconsin, United States) was used to measure the cellular viability during spore germination of different *Streptomyces* species, following the manufacturer’s instructions (Sun et al., 2016). This method detects cellular viability by measuring ATP content. According to the colony counting results of different species of *Streptomyces* during spore germination, RpfA protein of optimal concentration was added into spore suspension of optimal dilution gradient of the strain to be tested as the experimental group, and no RpfA protein was added as the control group, with three replicates each group. The cultures were incubated at 28°C with shaking at 160 rpm for 72 h. Luminescence intensity was measured every 12 h. Simultaneously, D_2_O isotope labeling combined with Single-Cell Raman Spectroscopy (D_2_O-SCRS) (Wang et al., 2022) was utilized to assess the metabolic activity of spore germination with and without the addition of RpfA protein.

### 2.5 Isolation and identification of *Streptomyces* species in frozen soils of the Qinghai-Tibet Plateau

Soil samples were collected from six locations in the frozen soils of the Qinghai-Tibet Plateau (Table 2). The processed soil samples were gradient-diluted to 10^−3^, and 100 μL of each gradient was plated onto R_2_A and glucose-tryptone solid media, respectively. The media were pre-supplemented with sterile-filtered *S. coelicolor* RpfA protein at concentrations of 10, 1, and 0.1% (v/v), as well as final concentrations of 50 μg/mL of cycloheximide, nalidixic acid, and nystatin, and 40 μg/mL of kanamycin. Groups without RpfA protein served as controls. Each group had three replicates, and they were incubated at 28°C for more than 2 weeks. The colonies on the plates were isolated, purified, and preserved.

**Table 2:** Distribution of sampling points.

Samples	Geographic coordinates	Altitude	Address	Characteristics
S1	28.323692919°N, 91.92733961°E	4757.0 m	Nari Yongco Lake, Cona County, Shannan City, Tibet Autonomous Region	Saltwater lake riparian zone
S2	28.95306387°N, 91.01763305°E	4686.4 m	Yamdrok Lake, Langkazi County, Shannan City, Tibet Autonomous Region	Freshwater lake riparian zone
S3	30.23657583°N, 90.64116536°E	4586.0 m	Damxung County, Lhasa City, Tibet Autonomous Region	Along the Qinghai-Tibet Railway
S4	30.85531513°N, 94.53199967°E	5293.0 m	Chagongla, Bianba County, Tibet Autonomous Region	Permafrost region
S5	31.72606427°N, 93.18113288°E	4705.0 m	Biru County, Nagqu City, Tibet Autonomous Region	Complex terrain
S6	30.88439766°N, 93.71450372°E	4807.0 m	Bianba County, Changdu City, Tibet Autonomous Region	Low temperature with large daily temperature variation

The Genomic DNA of the isolates were extracted using “chelex 100” method (de Lamballerie et al., 1992), and the 16S rRNA gene fragments were PCR-amplified using the universal bacterial primers 27F (5′-AGAGTTTGATCCTGGCTCAG-3′) and 1492R (5′-GGTTACCTTGTTACGACTT-3′). The amplification products were sent to Sangon Biotech (Shanghai) Co., Ltd. for sequencing. The sequencing results were analyzed and compared using the EzTaxon database on the EzBioCloud online website^4^ (Kim et al., 2012). Multiple alignments were performed using MEGA 11.0 (Kumar et al., 2016), and a phylogenetic tree was constructed using the Neighbor-Joining method (Saitou and Nei, 1987). The 16S rRNA gene trees diagram were drawn using the iTOL online tool^5^ (Lefebvre et al., 2009) to determine the taxonomic status and affiliation of the strains.

### 2.6 Statistical analysis

Statistical analysis of the data was performed using GraphPad Prism (version 9.4.1) and Origin 8.0. The mean ± standard deviation (SD) for each parameter was calculated using data from three replicates. The significance level for analysis of variance (ANOVA) was set at *p* < 0.05.

## 3 Results

### 3.1 Characteristics of the RpfA protein from *Streptomyces coelicolor* CGMCC 4.1658^T^

**Figure 1:**
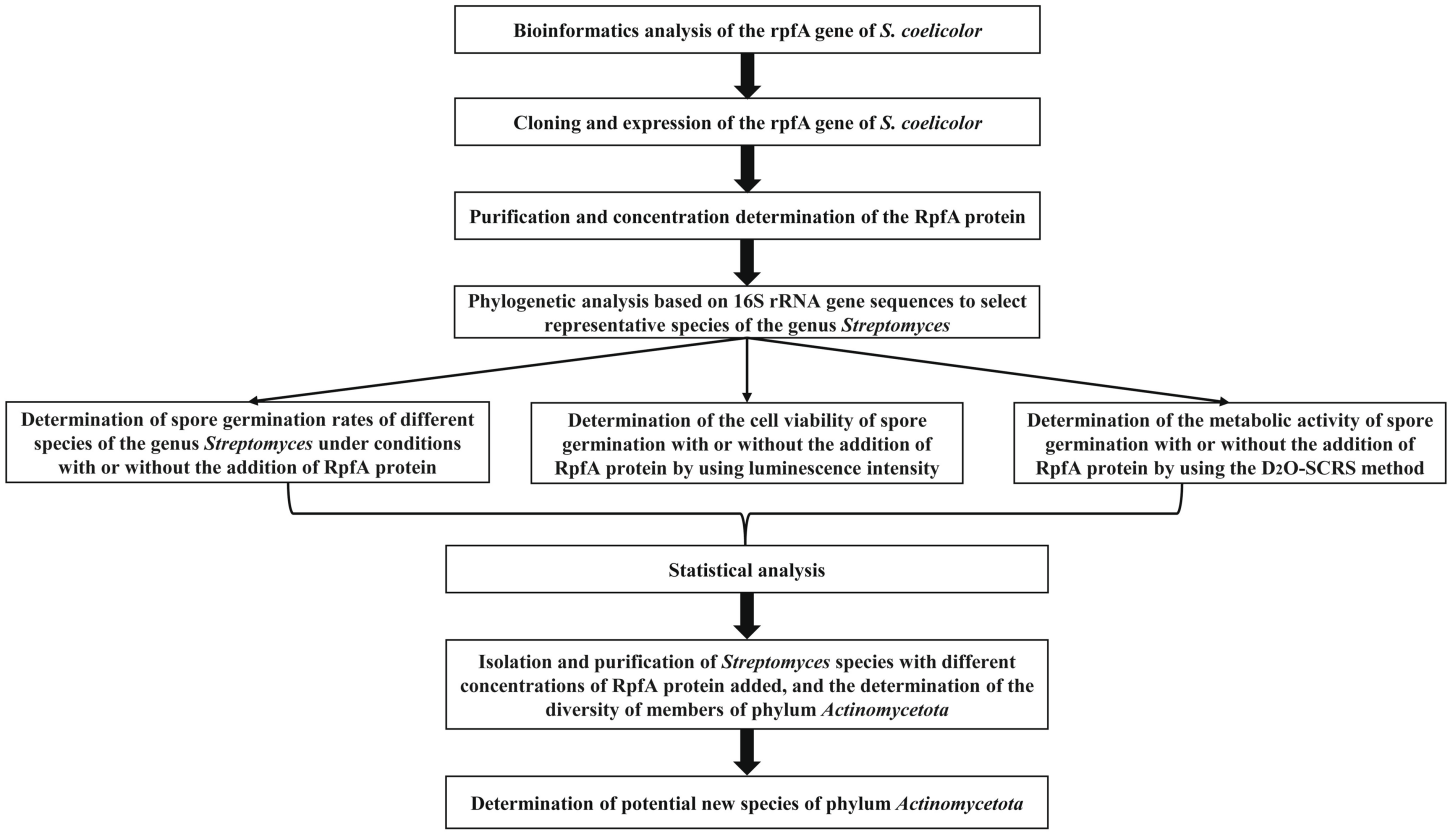
The flowchart of the experimental scheme of this study.

**Figure 2:**
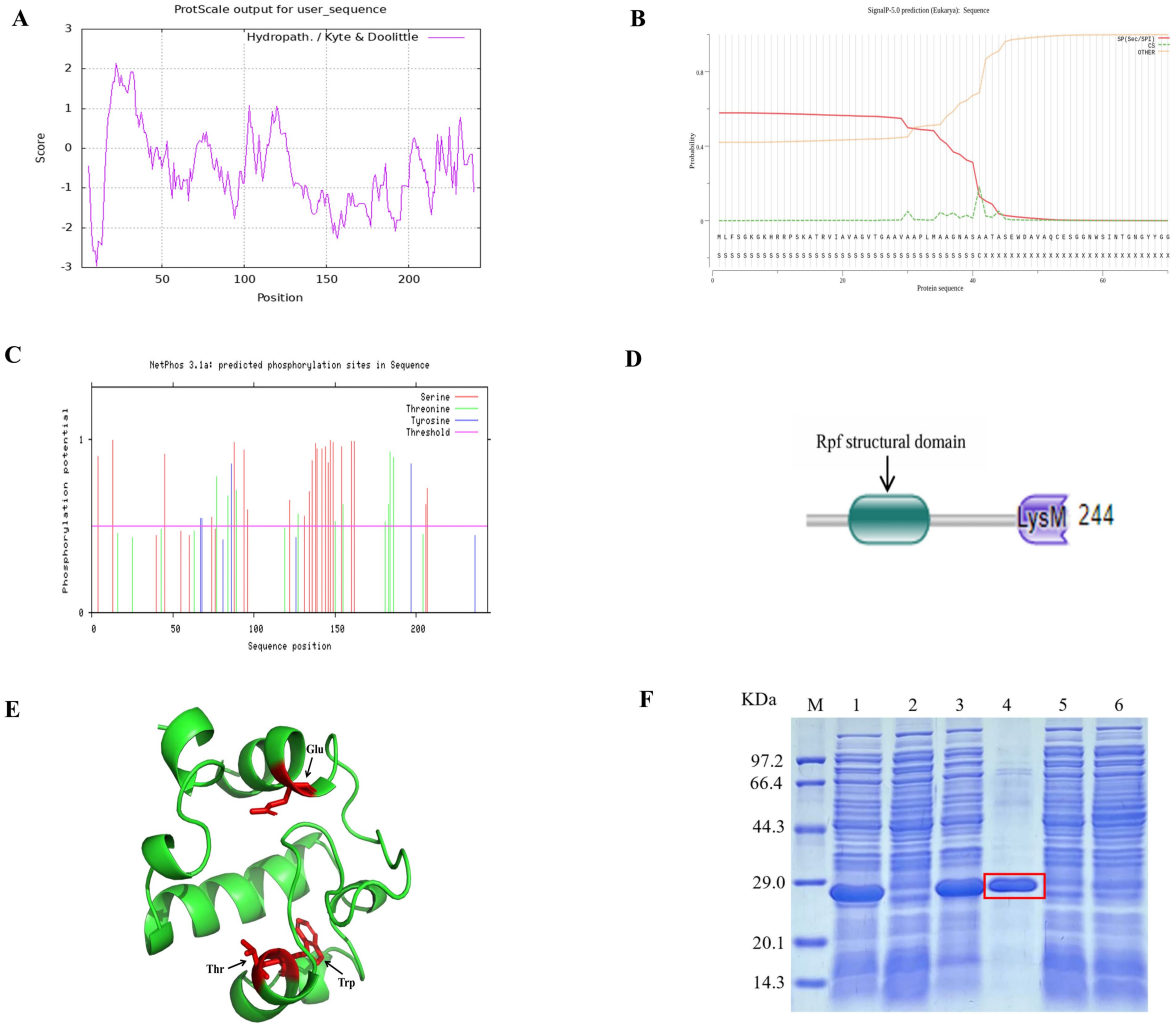
Characteristic information of the RpfA protein from *S. coelicolor*. **(A)** Hydrophilicity and hydrophobicity prediction diagram; **(B)** Signal peptide prediction diagram; **(C)** Phosphorylation site prediction diagram; **(D)** Structural domains; **(E)** Tertiary structure prediction diagram, Glu is the key catalytic site of the RpfA protein in *S.coelicolor*, Thr and Try are its substrate-binding residues; **(F)** SDS-PAGE electrophoresis for detection of *rpfA* gene expression and protein purification. M: Protein molecular weight markers; Lane 1 and Lane 3: lysed proteins of *E. coli* BL21(DE3) containing pET-28a (+)-*rpf*; Lane 2 and Lane 5: lysed proteins of *E. coli* BL21(DE3) containing pET-28a (+); Lane 4: purified RpfA protein (marked with a red frame); Lane 6: lysed proteins of *E. coli* BL21(DE3) containing pET-28a (+)-*rpf* before induction.

The experimental scheme of this study is shown in Figure 1. The physicochemical properties of the amino acid sequence encoded by the *rpfA* gene of *S. coelicolor* were predicted and analyzed using the Expasy online software.^6^ The protein consists of 244 amino acids, including 20 types of amino acids, with alanine (Ala) and glycine (Gly) being the most abundant. The Grand average of hydropathicity (GRAVY) is −0.519. The II value of this protein is 25.39. The hydrophilicity and hydrophobicity of the RpfA protein amino acid sequence were analyzed using the ProtScale tool. The hydrophilic amino acids in the RpfA protein amino acid sequence are slightly more abundant than the hydrophobic amino acids, and the amino acid hydrophilicity index is around −1 (Figure 2A). Prediction and analysis of the signal peptide of the RpfA protein encoded by the *rpfA* gene was conducted using the online software Signal P-5.0.^7^ The possibility of the cleavage site of the signal peptide of the RpfA protein being located between the 41st and 42nd amino acids (ASA-AT) is 0.1830, and the possibility of the presence of a Sec/SPI signal peptide is 0.5828 (Figure 2B). The amino acid sequence of the RpfA protein was analyzed for potential phosphorylation sites using the online tool NetPhos 3.1.^8^ The analysis results indicated that the RpfA protein has 23 serine (Ser) phosphorylation sites, 10 threonine (Thr) phosphorylation sites, and 4 tyrosine (Tyr) phosphorylation sites (Figure 2C). The conserved domains of the protein sequence encoded by the *rpfA* gene were predicted and analyzed using HM-MER.^9^ The RpfA protein-encoding chain has two domains: one Rpf catalytic domain for hydrolyzing β-1,4-glycosidic bonds and one Lysin (LysM, C121525) domain (Figure 2D). The three-dimensional structure of the RpfA protein was predicted using SWISS-MODEL (Waterhouse et al., 2018). The results showed that its key catalytic site was glutamic acid, the substrate-binding residues were threonine and tryptophan. The reliability score of the RpfA protein is 57.69%. The template sequence method used X-ray with a resolution of 1.17 Å. The alignment covered amino acids 45–122, with a coverage of 0.32 (Figure 2E).

### 3.2 Purification and identification of the RpfA protein from *Streptomyces coelicolor* CGMCC 4.1658^T^

The prokaryotic expression vector pET-28a (+)-*rpfA* for the *rpfA* gene of *S. coelicolor* was constructed and efficiently expressed heterologously in *E. coli* BL21 (DE3). The heterologously expressed RpfA protein was purified using Ni-NTA-agarose affinity chromatography, yielding RpfA protein with a molecular weight of approximately 24.9 kDa and a concentration of 1.19 mg/mL (Figure 2F).

### 3.3 Phylogenetic analysis of some species within the genus *Streptomyces* and selection of representative species

Sixty type strains of *Streptomyces* species (Supplementary Table S1) formed five major phylogenetic groups (Supplementary Figure S1). One representative species was selected from each group (indicated in bold), and they were *S. coelicolor* CGMCC 4.1658^T^, *S. griseus* CGMCC 4.1419^T^, *S. laurentii* JCM 5063^T^, *S. niveus* JCM 4251^T^, and *S. lusitanus* HBU208066^T^, respectively. These strains were used as test strains in subsequent experiments to verify the ability of the RpfA protein from *S. coelicolor* to promote spore germination activity in itself and other *Streptomyces* species.

### 3.4 Effect of RpfA protein on spore germination rates among different *Streptomyces* species

**Figure 3:**
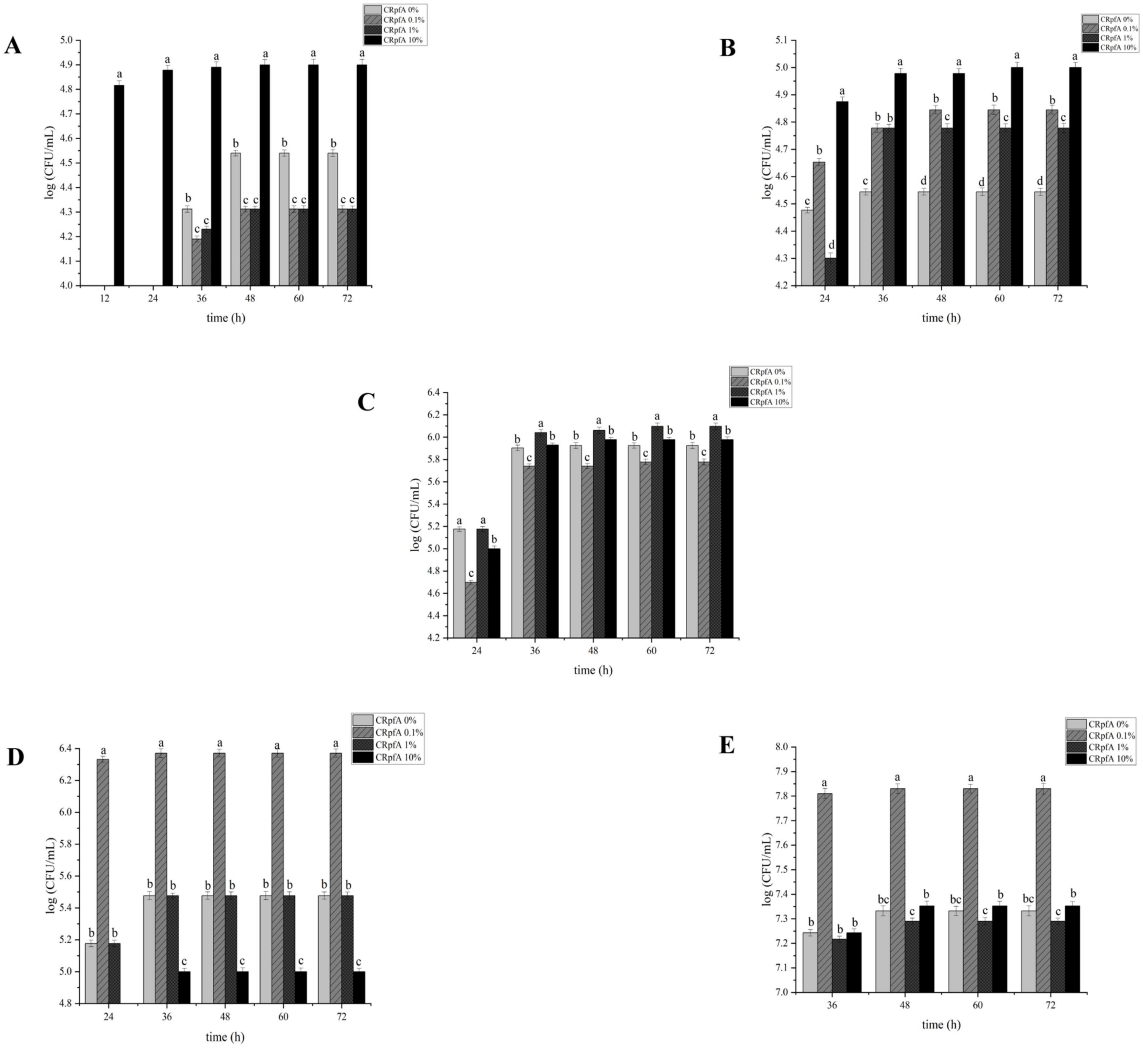
Effect of different concentrations of RpfA protein on spore germination of *Streptomyces* strain. Different letters above the bar graph indicate statistically significant differences at *p* ≤ 0.05 (*n* = 3). **(A)** *S. coelicolor*; **(B)** *S. niveus*; **(C)** *S. griseus*; **(D)** *S. laurentii*; **(E)** *S. lusitanus*; CRpfA represents the concentration of RpfA protein.

**Figure 4:**
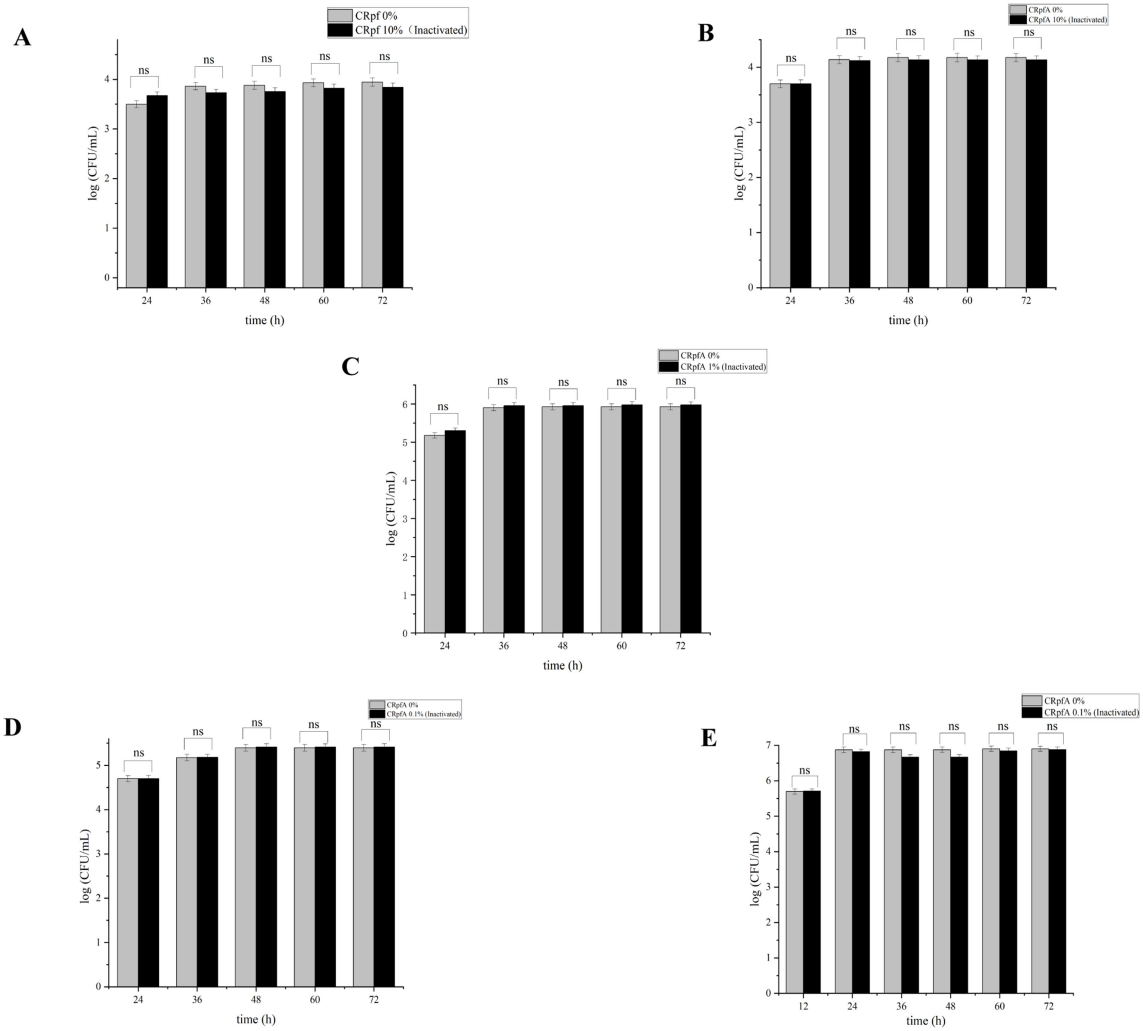
Effect of inactivated RpfA protein on the number of colonies formed by *Streptomyces* spore germination. Significance level of ANOVA, *(0.01 < =*p* < 0.05), **(0.001 < =*p* < 0.01), ***(*p* < 0.001), ns(*p* > 0.05), (*n* = 3). **(A)** *S. coelicolor*; **(B)** *S. niveus*; **(C)** *S. griseus*; **(D)** *S. laurentii*; **(E)** *S. lusitanus*; CRpfA represents the concentration of RpfA protein.

The addition of different concentrations of RpfA protein had varying effects on the spore germination rates of *S. coelicolor*, *S. griseus*, *S. laurentii*, *S. niveus*, and *S. lusitanus* (Figure 3). When a final concentration of 10% (v/v) RpfA protein was added to R_2_A medium, the number of colonies formed by the spores of *S. coelicolor* and *S. niveus* was significantly higher than that in the experimental groups with 0.1 and 1% (v/v) RpfA protein added and the control group without RpfA protein. The addition of 1% (v/v) RpfA protein had the most significant promoting effect on spore germination of *S. griseus*. In contrast, 0.1% (v/v) RpfA protein had the most significant promoting effect on spore germination of *S. laurentii* and *S. lusitanus*. After adding different concentrations of RpfA protein, the spore germination rates of different *Streptomyces* species varied (Supplementary Table S2). Compared with the control group, when cultured for 72 h and after adding the optimal concentration of RpfA protein, the spore germination rates of *S. coelicolor*, *S. niveus*, *S. griseus*, *S. laurentii*, and *S. lusitanus* increased by 45, 48.9, 22.2, 64, and 48.9%, respectively (Table 3). Furthermore, it was found that inactivated RpfA protein did not promote spore germination (Figure 4).

**Table 3:** Spore germination rates of different *Streptomyces* species in the experimental group with the optimal concentration of Rpf protein and the control group at 72 h of cultivation.

Species	Initial spore concentration (CFU/mL)	−RpfA (CFU/mL)	Spore germination rate	+RpfA (CFU/mL)	Spore germination rate	Increase in spore germination rate
*S. coelicolor*	(1.01 ± 0.2) × 10^6^	(3.45 ± 0.4) × 10^5b^	34.2%	(8.0 ± 0.7) × 10^5a^	79.2%	45%
*S. niveus*	(1.2 ± 0.1) × 10^5^	(3.5 ± 0.3) × 10^4b^	29.2%	(1.0 ± 0.3) × 10^5a^	83.3%	54.1%
*S. griseus*	(1.8 ± 0.3) × 10^6^	(8.5 ± 0.2) × 10^5b^	47.2%	(1.25 ± 0.3) × 10^6a^	69.4%	22.2%
*S. laurentii*	(3.2 ± 0.2) × 10^6^	(3.0 ± 0.1) × 10^5b^	9.4%	(2.35 ± 0.1) × 10^6a^	73.4%	64%
*S. lusitanus*	(9.4 ± 0.5) × 10^7^	(2.15 ± 0.1) × 10^7b^	22.9%	6.75 ± 0.4 × 10^7a^	71.8%	48.9%

Different letters indicate statistically significant differences at *p* ≤ 0.05 (*n* = 3); “−RpfA” and “+RpfA” represent the absence and presence of RpfA protein, respectively.

### 3.5 Cell viability during spore germination measured by luminescence intensity

**Figure 5:**
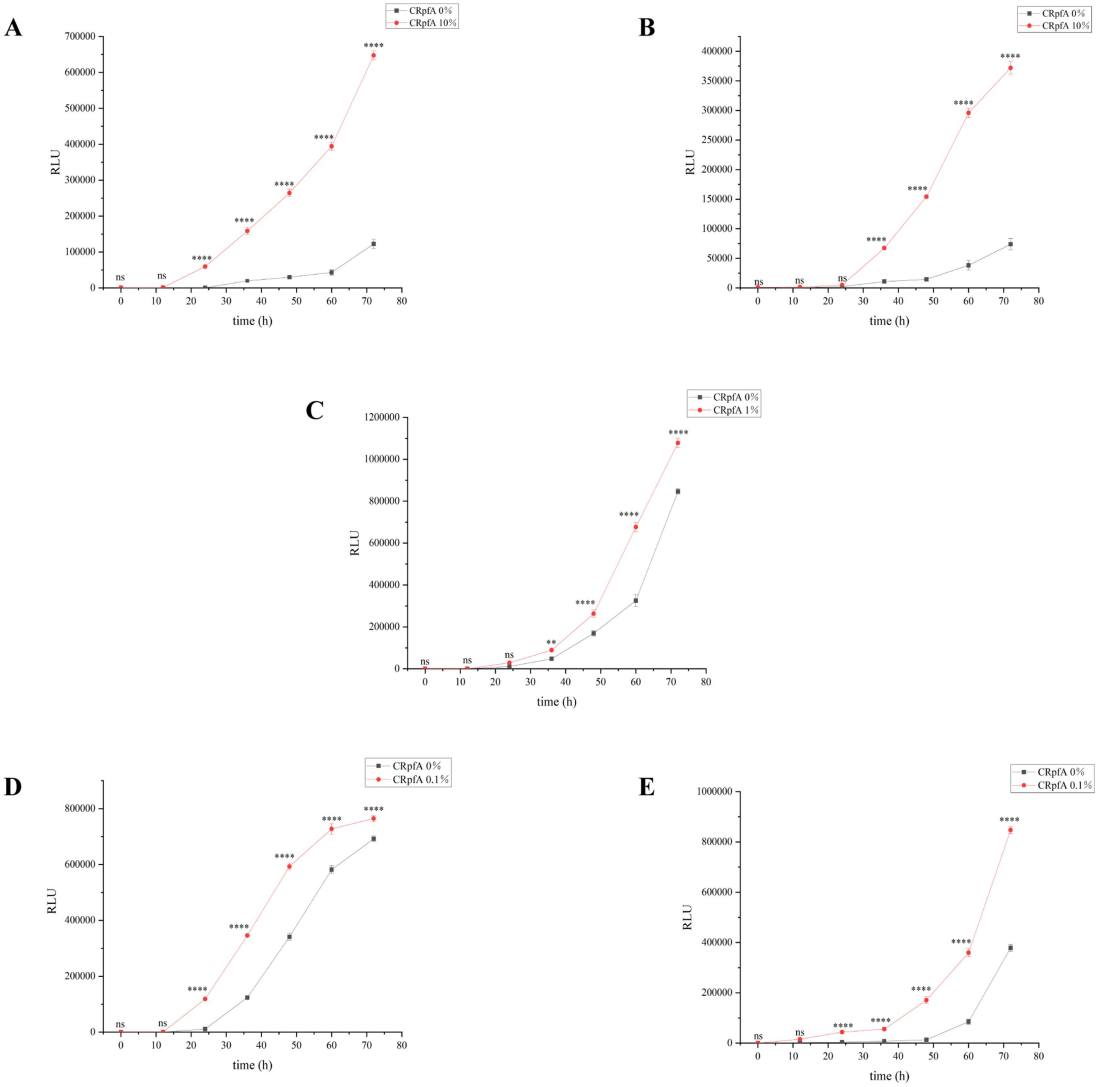
Verification of the effect of RpfA protein on cellular viability during *Streptomyces* spore germination using BacTiter-Glo™. Significance level of ANOVA, *(0.01 < =*p* < 0.05), **(0.001 < =*p* < 0.01), ***(*p* < 0.001), ns(*p* > 0.05), (*n* = 3). **(A)** *S. coelicolor*; **(B)** *S. niveus*; **(C)** *S. griseus*; **(D)** *S. laurentii*; **(E)** *S. lusitanus*; CRpfA represents the concentration of RpfA protein.

To further verify the promoting effect of RpfA protein on spore germination, the BacTiter-Glo™ Microbial Cell Viability Assay Kit was used to measure the cell viability during spore germination of *S. coelicolor* CGMCC 4.1658^T^, *S. niveus* JCM 4251^T^, *S. griseus* CGMCC 4.1419^T^, *S. laurentii* JCM 5063^T^, and *S. lusitanus* HBU208066^T^ in the presence or absence of RpfA protein (Figure 5). The BacTiter-Glo™ reagent relies on the properties of thermostable luciferase (Ultra-Glo recombinant luciferase) and a patented formula for extracting ATP from bacteria. The BacTiter-Glo™ assay produces a luminescent signal, with the intensity of the signal being directly proportional to the amount of ATP present, which in turn is proportional to the cellular viability in the culture. Bioluminescent sensors based on luciferin have been proven to effectively detect ATP concentrations, with a linear relationship between analyte concentration and bioluminescent intensity (Abbasi et al., 2024; Karimi et al., 2022; Luo et al., 2009). The assay results showed that, compared with the control group, the experimental group with the optimal concentration of RpfA protein had continuously increasing Relative Light Unit (RLU) values over time, which were significantly higher than those of the control group.

### 3.6 Metabolic activity of spores during germination determined by D_2_O isotope labeling combined with single-cell Raman spectroscopy (D_2_O-SCRS)

Raman spectroscopy is a fingerprint spectrum that reflects the structure of materials, arising from the interaction between light and material. When high-intensity incident light scatters on material molecules, a portion of the scattered light differs in frequency from the incident light, with the frequency shifting based on the structure of the tested material. This portion of the scattered light is known as Raman scattering (Huang et al., 2010; Smith et al., 2016). The use of heavy water (D_2_O) isotope labeling combined with single-cell Raman spectroscopy (SCRS) allows for the identification and classification of viable microorganisms at the single-cell level in complex samples. The carbon-deuterium (C-D) characteristic peak in single-cell Raman spectroscopy can clearly detect the incorporation of D, derived from D_2_O, into the biomass of microorganisms, thereby reflecting their metabolic activity (Berry et al., 2015; Hua et al., 2021; Ni et al., 2021).

**Figure 6:**
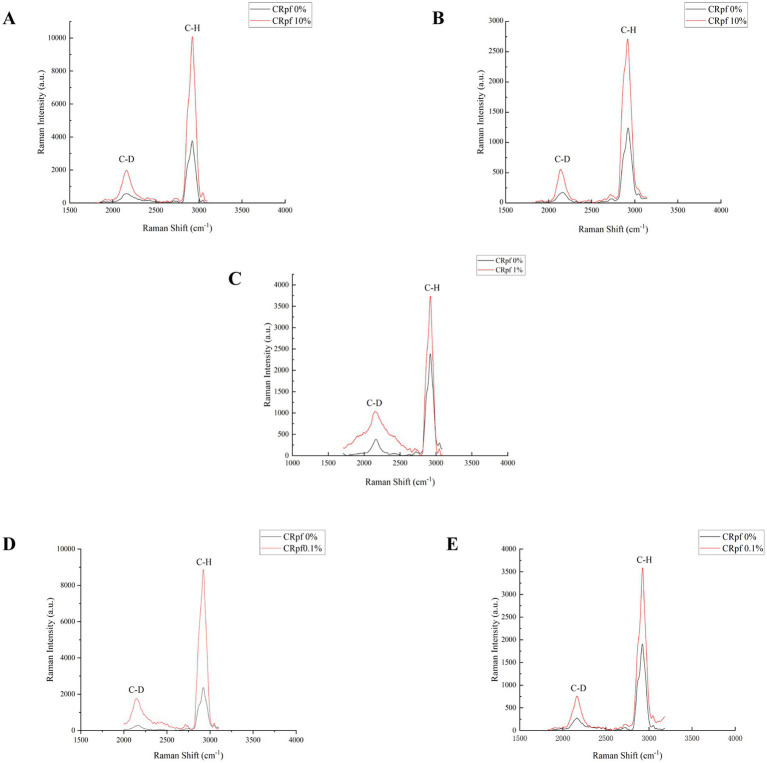
Peak of single-cell Raman spectroscopy during spore germination after D_2_O isotope labeling. **(A)** *S. coelicolor*; **(B)** *S. niveus*; **(C)** *S. griseus*; **(D)** *S. laurentii*; **(E)** *S. lusitanus*; CRpfA represents the concentration of RpfA protein.

Raman spectroscopy was adopted to further verify the effect of RpfA protein on the metabolic activity during spore germination of *S. coelicolor* CGMCC 4.1658^T^, *S. niveus* JCM 4251^T^, *S. griseus* CGMCC 4.1419^T^, *S. laurentii* JCM 5063^T^, and S. lusitanus HBU208066T (Figure 6). After 72 h of culturing, the intensities of the C-D and C-H peaks of the prepared samples were detected by a single-cell Raman phenotypic monitoring system, and their C-D values were calculated (Table 4). The results showed that in the experimental groups with the optimal concentration of RpfA protein added, the C-D values of all five *Streptomyces* strains were higher than those in the control group to varying degrees, indicating that the cellular metabolic activity in the experimental groups with RpfA protein during spore germination was higher. Additionally, both the C-D and C-H peak intensities were higher in the experimental groups.

**Table 4:** C–D values (%) of each *Streptomyces* species in the experimental and control groups.

Species	C-D value (%)	
	−RpfA	+RpfA
*S. coelicolor*	19.1	20.9
*S. niveus*	13.9	16.7
*S. griseus*	16.0	35.1
*S. laurentii*	12.6	23.4
*S. lusitanus*	16.3	20.2

“−RpfA” and “+RpfA” represent the absence and presence of RpfA protein, respectively.

### 3.7 *Streptomyces* strains isolated from six soil samples after adding RpfA protein

From soil samples collected at six sampling sites in the frozen soils of the Qinghai-Tibet Plateau, a total of 137 strains of *Streptomyces* were isolated using R_2_A and glucose-tryptone media (including experimental groups with different concentrations of RpfA protein added and control groups without RpfA protein added). Specifically, 80, 27, and 25 strains of *Streptomyces* were obtained when the concentrations of RpfA protein added were 10, 1, and 0.1%, respectively; whereas in the control groups without RpfA protein added, only 5 strains of *Streptomyces* were isolated (Table 5).

**Table 5:** The quantities of strains of genus *Streptomyces* isolated by adding different the concentrations of RpfA protein in six soil samples.

Samples	Numbers of strains of *Streptomyces*			
	CRpfA 0%	CRpfA 0.1%	CRpfA 1%	CRpfA 10%
S1	1	7	4	19
S2	0	4	5	26
S3	2	7	8	16
S4	0	4	5	7
S5	1	3	3	10
S6	1	0	2	2
Total	5	25	27	80

CRpfA represents the concentration of RpfA protein.

When 10, 1, and 0.1% RpfA protein were added to the media, 19, 4, and 7 strains of genus *Streptomyces* were isolated from sample S1, respectively; while only 1 strain was isolated in the control group. In sample S2, 26, 5, and 4 strains were obtained, respectively, with no *Streptomyces* strains isolated in the control group. For sample S3, 16, 8, and 7 strains were isolated, respectively, while 2 strains were isolated in the control group. In sample S4, 7, 5, and 4 strains were obtained, respectively, with no *Streptomyces* strains isolated in the control group. For sample S5, 10, 3, and 3 strains were isolated, respectively, while only 1 strain was isolated in the control group. In sample S6, 2 strains of *Streptomyces* were isolated when both 10 and 1% RpfA protein were added, but no *Streptomyces* strains were isolated when 0.1% RpfA protein was added; 1 strain was isolated in the control group (Table 5).

**Figure 7:**
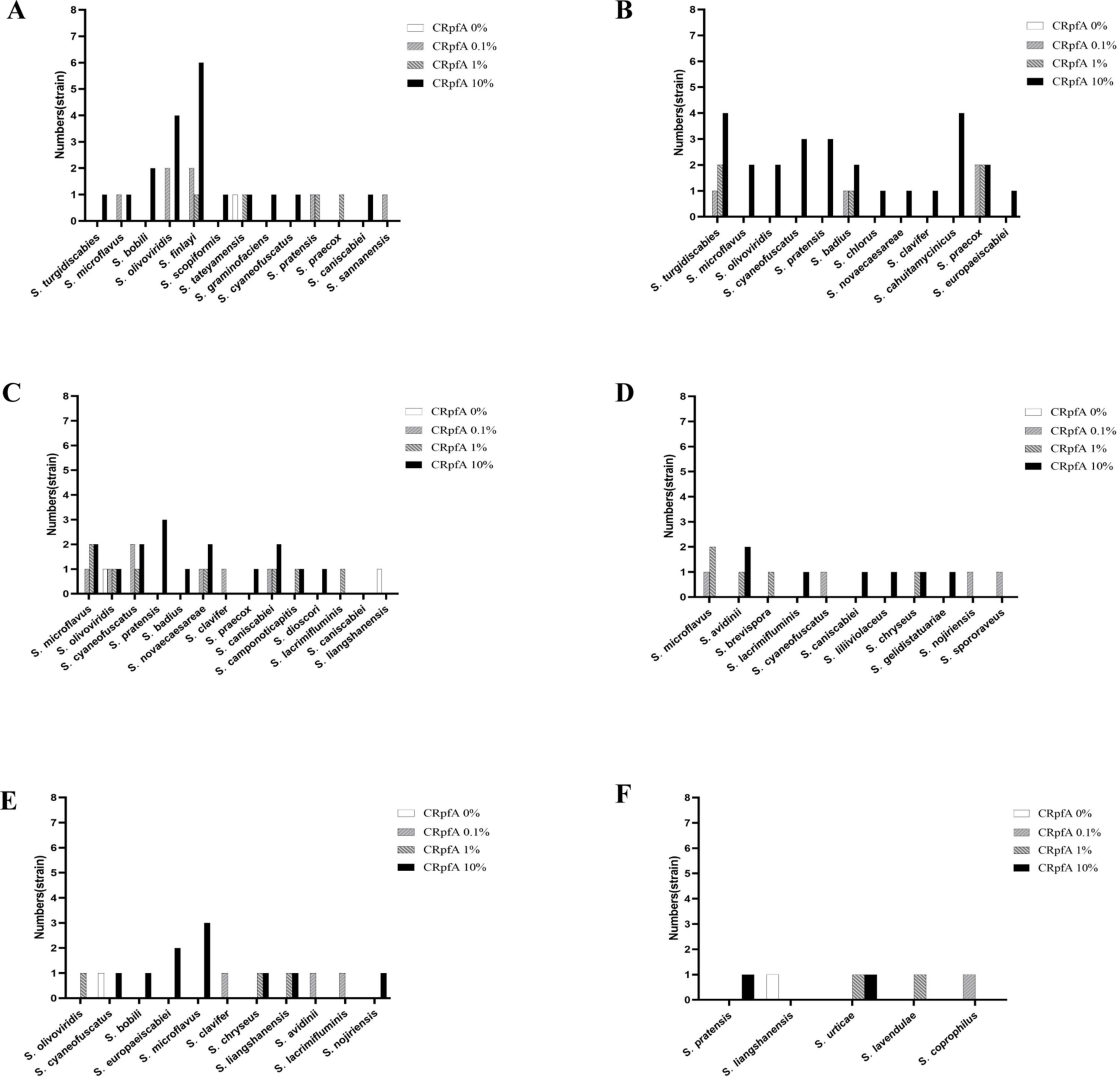
Statistical graphs of the quantities of *Streptomyces* strains isolated from six soil samples adding different the concentrations of RpfA protein. **(A)** S1; **(B)** S2; **(C)** S3; **(D)** S4; **(E)** S5; **(F)** S6; CRpfA represents the concentration of RpfA protein.

16S rRNA sequence analysis was conducted on all *Streptomyces* strains isolated from the six samples to determine their species (Supplementary Table S3). The results revealed that the addition of RpfA protein in the six samples increased the diversity and quantity of isolated *Streptomyces* to varying degrees (Supplementary Tables S4–S9; Figure 7). Among the five *Streptomyces* strains isolated from the control groups without RpfA protein, they belonged to four species: *S. tateyamensis*, *S. olivoviridis*, *S. liangshanensis*, and *S. cyaneofuscatus*. Except for *S. liangshanensis*, the other three species were also isolated in the experimental groups with RpfA protein added.

**Figure 8:**
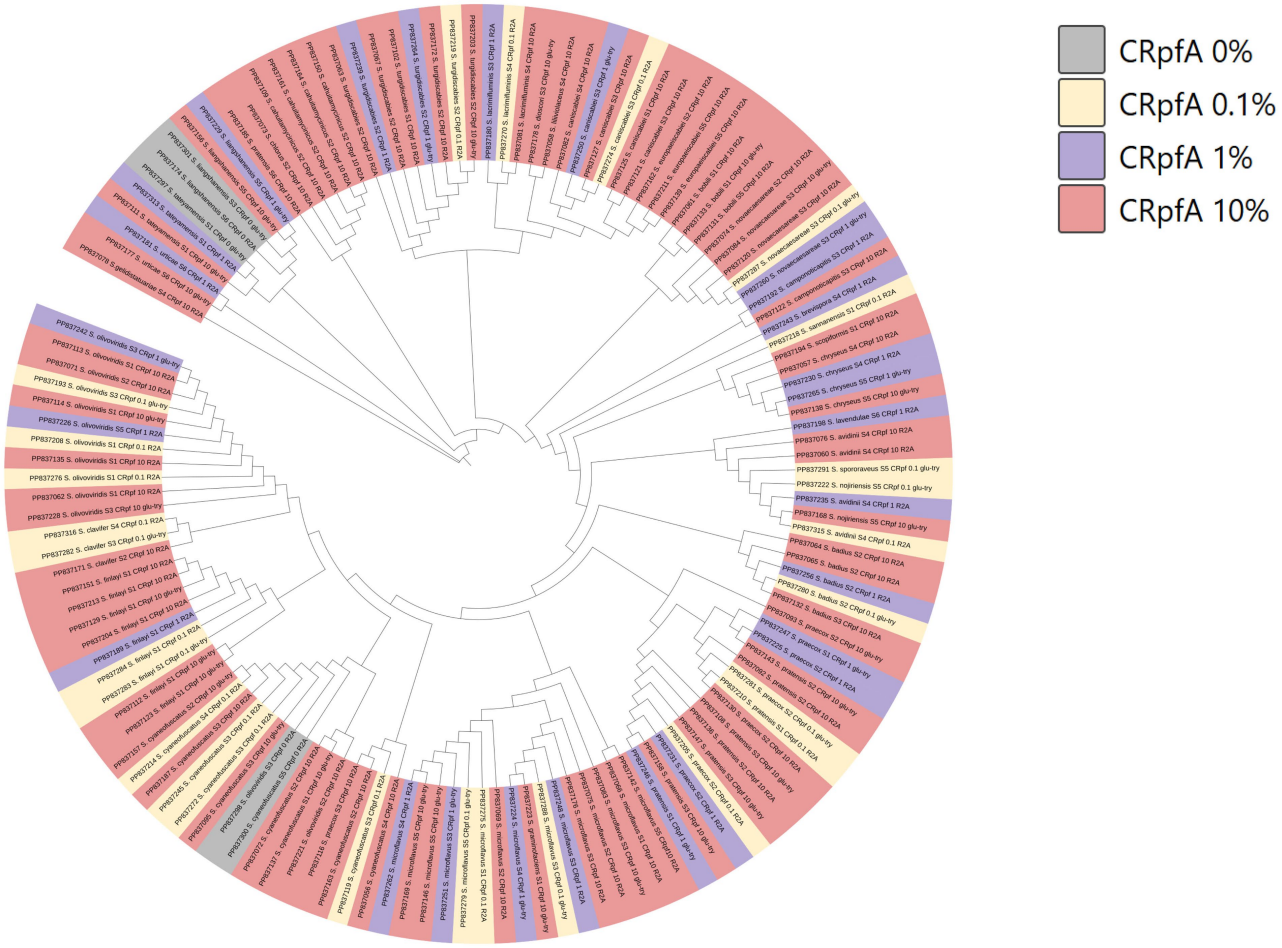
Phylogenetic tree constructed based on *Streptomyces* strains isolated from six samples. CRpfA represents the concentration of RpfA protein.

Based on the 16S rRNA gene sequences of these 137 *Streptomyces* strains, a phylogenetic tree was constructed (Figure 8). It was found that the five *Streptomyces* strains isolated from the control groups without RpfA protein addition clustered into two small groups.

### 3.8 Diversity of members of phylum *Actinomycetota* obtained by adding RpfA protein

**Figure 9:**
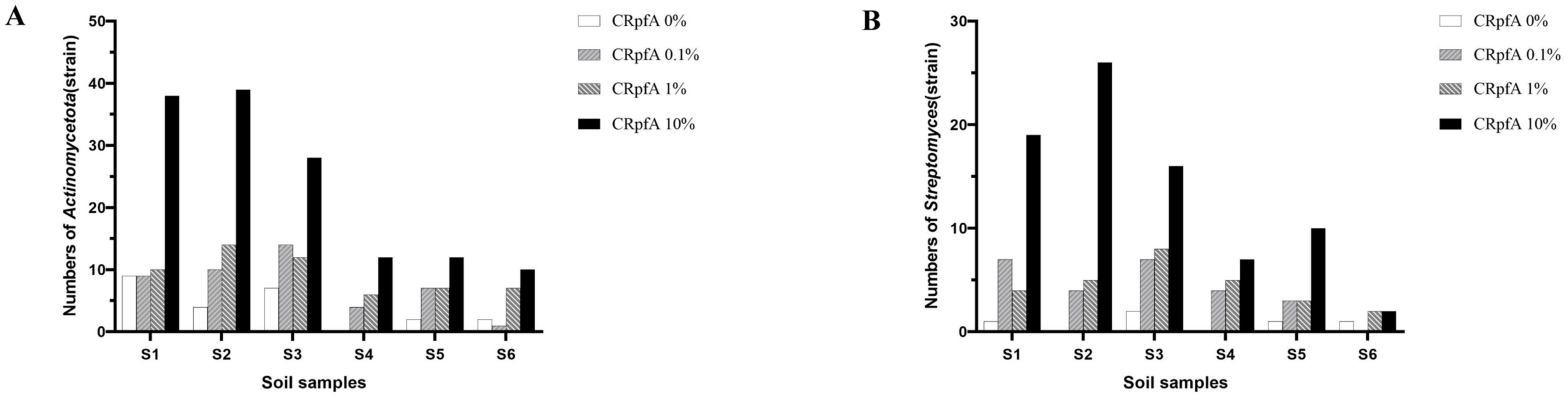
Statistical graphs of the quantities of strains of phylum *Actinomycetota* **(A)** and genus *Streptomyces* **(B)** strains isolated from six soil samples. CRpfA represents the concentration of RpfA protein.

**Figure 10:**
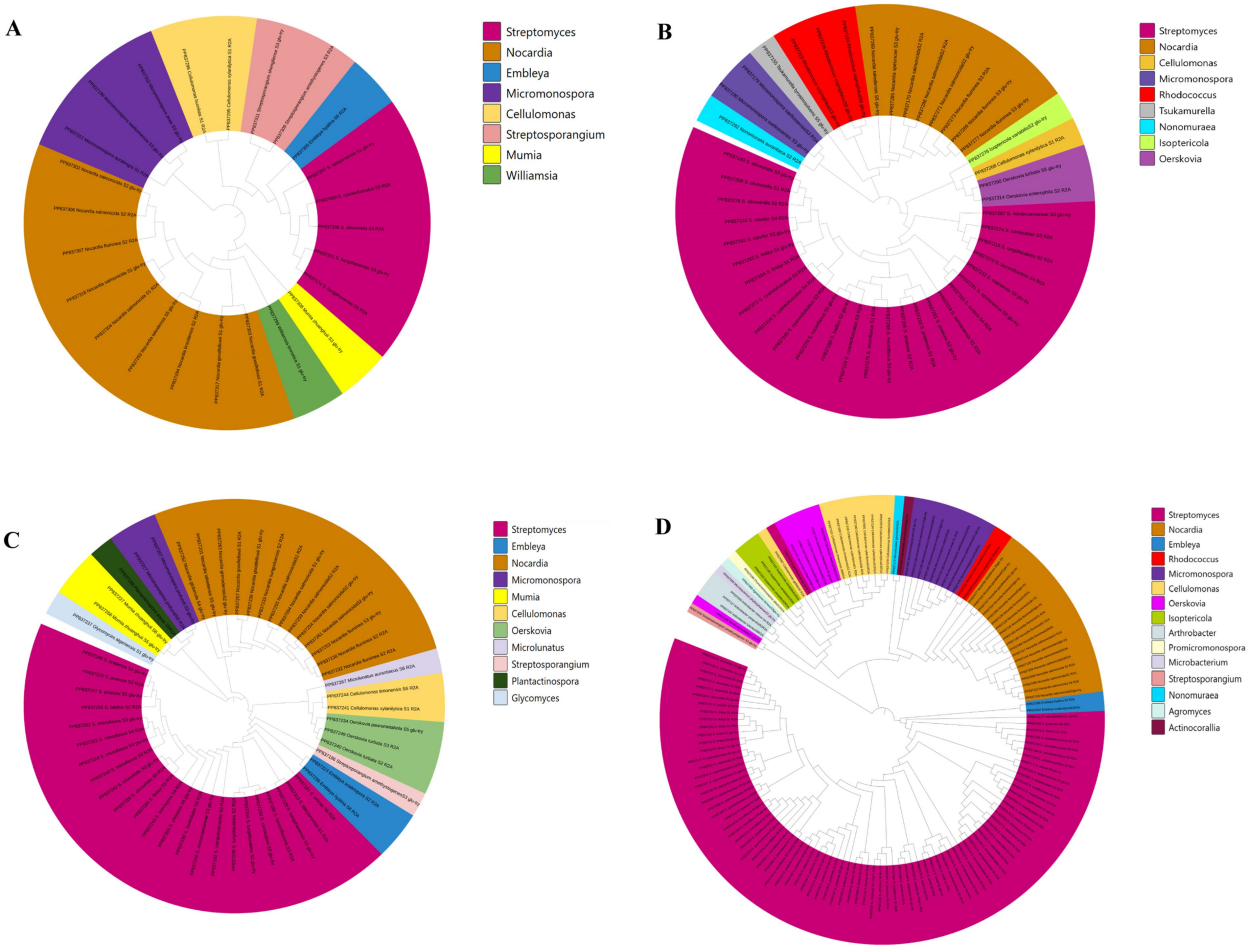
Phylogenetic tree constructed based on phylum *Actinomycetota* strains isolated from the frozen soils of the Qinghai-Tibet Plateau. **(A)** CRpfA 0%; **(B)** CRpfA 0.1%; **(C)** CRpfA 1%; **(D)** CRpfA 10%; CRpfA represents the concentration of RpfA protein.

A total of 264 strains (Supplementary Tables S3, S10) of phylum *Actinomycetotas* were isolated from six soil samples collected from the frozen soils of the Qinghai-Tibet Plateau (Figure 9). The quantities and proportions different genera of phylum *Actinomycetota* obtained by adding different concentrations of RpfA protein are shown in Supplementary Table S11, and a phylogenetic tree was constructed based on these strains (Figure 10). When 10% RpfA protein was added, a total of 139 strains of *Actinomycetota*, belonging to 15 genera, were isolated, with *Streptomyces* being the dominant genus, accounting for 80 strains and 57.55% of the total. When 1% RpfA protein was added, 56 strains of phylum *Actinomycetota*, belonging to 11 genera, were isolated, with *Streptomyces* also being the dominant genus, accounting for 27 strains and 48.21% of the total. When 0.1% RpfA protein was added, 45 strains of phylum *Actinomycetota*, belonging to 9 genera, were isolated, with *Streptomyces* again being the dominant genus, accounting for 25 strains and 55.56% of the total. However, in the control group without RpfA protein, 24 strains of phylum *Actinomycetota*, belonging to 8 genera, were isolated, with *Nocardia* being the dominant genus, accounting for 9 strains and 37.50% of the total, while only 5 *Streptomyces* strains were isolated. The diversity of the members of phylum *Actinomycetota* isolated from the 6 samples with the addition of different concentrations of RpfA protein using R_2_A and glucose-tryptone media at the generic level was shown in Supplementary Figure S2.

Furthermore, it was also found that five genera were only isolated when 10% RpfA protein was added, namely *Arthrobacter* (2.16%), *Actinocorallia* (0.72%), *Agromyces* (0.72%), *Microbacterium* (0.72%) and *Promicromonospora* (0.72%). Three genera were only isolated when 1% RpfA protein was added, namely *Glycomyces* (1.79%), *Microlunatus* (1.79%), and *Plantactinospora* (1.79%). One genus, *Tsukamurella* (2.22%), was only isolated when the concentration of RpfA protein added was 0.1%. In contrast, *Williamsia* (4.17%) was only isolated in the absence of RpfA protein.

### 3.9 Potential new species of phylum *Actinomycetota*

Among the isolated strains of phylum *Actinomycetota*, a total of 21 strains exhibited <98.65% similarity in their 16S rRNA gene sequences to the closest type strains of their relatives, falling below the threshold for species delineation in prokaryotes (Kim et al., 2014). These were considered potential new species, accounting for 15.3% of the isolated strains, including 11 within the genus *Streptomyces* and 10 in other genera (Supplementary Table S12). In the R_2_A medium, when 10% RpfA protein is added, 6 potential new species of the *Streptomyces* genus, 2 potential new species of the *Cellulomonas* genus, and 1 potential new species of the *Embleya* genus are isolated; when 1% RpfA protein is added, 2 potential new species of the *Streptomyces* genus and 1 potential new species of the *Embleya* genus are isolated; when 0.1% RpfA protein is added, 1 potential new species of the *Streptomyces* genus, 1 potential new species of the *Cellulomonas* genus, and 1 potential new species of the *Nonomuraea* genus are isolated; when no RpfA protein is added, 1 potential new species of the *Nocardia* genus and 1 potential new species of the *Embleya* genus are isolated. In the glucose-tryptone medium, when 10% RpfA protein is added, 1 potential new species of the *Streptomyces* genus and 1 potential new species of the *Micromonospora* genus are isolated; when no RpfA protein is added, 1 potential new species of the *Streptomyces* genus and 1 potential new species of the *Williamsia* genus are isolated.

## 4 Discussion

In this study, bioinformatics method was used to predict the structure and related information of the RpfA protein of *S. coelicolor.* The instability index (II) value of this protein is 25.39. Generally, a protein with an II value less than 40 is considered to be stable (Gamage et al., 2019), indicating that this protein is relatively stable. According to the analysis results of the physicochemical properties of the amino acid sequence encoded by the *rpfA* gene of *S. coelicolor*, the hydrophilic amino acids in the RpfA protein amino acid sequence are slightly more than the hydrophobic amino acids, and the amino acid hydrophilicity index is around −1, indicating that the RpfA protein is a hydrophilic protein. The prediction and analysis by the online software Signal P-5.0 show that RpfA has a signal peptide. The SP (Sec/SPI) curve indicates that RpfA is a typical secretory protein. The signal peptide binds to receptors on the cell membrane, guiding the transport of proteins and closely related to the soluble expression of proteins (Gong et al., 2022). Therefore, it is speculated that the RpfA protein can be expressed in a soluble form. The RpfA protein has 23 serine (Ser) phosphorylation sites, 10 threonine (Thr) phosphorylation sites, and 4 tyrosine (Tyr) phosphorylation sites (Figure 1C). It is speculated that the RpfA protein may participate in cell wall activities through reversible phosphorylation, which is thought to further promote to cell resuscitation (Cohen-Gonsaud et al., 2005; Cohen-Gonsaud et al., 2004). The RpfA protein-encoding chain has two domains: one Rpf catalytic domain for hydrolyzing β-1,4-glycosidic bonds and one Lysin (LysM, C121525) domain related to the properties of lysozyme (Buist et al., 2008). The homologous modeling results of *S. coelicolor rpfA* gene showed that the RpfA protein had the highest homology with *M. tuberculosis rpfB*, and the sequence matching degree was 49% (<70% was identified as a new protein), which contained a complete domain (Nikitushkin et al., 2015). The RpfA protein of *S. coelicolor* has a glycosylase - like domain in structure, with glutamic acid as its key catalytic site and threonine and tryptophan as the substrate - binding residues. These results lay a foundation for the subsequent research work and are helpful to further reveal the biological function of this protein in *S. coelicolor*.

In this study, the *rpfA* gene from *S. coelicolor* was heterologously expressed in *E. coli*, resulting in the production of recombinant RpfA protein with a molecular weight of approximately 24.9 kDa. The effect of the purified RpfA protein on the spore germination rate of different species of the genus *Streptomyces* was determined. The RpfA protein can promote the germination of its own spores as well as those of other *Streptomyces* species. Furthermore, it was found that inactivated RpfA protein did not promote spore germination (Figure 4), further proving that RpfA protein promotes spore germination not as a nutrient but as a protein with resuscitation function. It has also been shown that the products encoded by the five *rpf* genes (*rpfA-E*) of *S. coelicolor* can promote the rapid germination of dormant spores and affect the vegetative cgrowth and sporulation (Haiser et al., 2009; Sexton et al., 2015), which is consistent with the above results. The effect of RpfA protein on the viability of spore germination cells was determined using the BacTiter-Glo™ Microbial Cell Viability Assay Kit, and the assay results showed that, compared with the control group, the experimental group with the optimal concentration of RpfA protein had continuously increasing RLU values over time, which were significantly higher than those of the control group. This indicated that after adding RpfA protein, the cell viability during spore germination was more vigorous and the metabolic level was higher, further proving that the RpfA protein of *S. coelicolor* can promote the germination of its own spores as well as those of other *Streptomyces* species. The optimal concentration of this RpfA protein for promoting spore germination varies among species, and not always is a higher concentration of RpfA protein associated with a better effect on spore germination. It is speculated that this difference in activity and concentration may be related to differences in the genome and growth cycle of different *Streptomyces* species (Flärdh and Buttner, 2009), as well as the copy number, length of the *rpfs* gene, and genetic diversity of Rpf proteins (Tantivitayakul et al., 2020). This hypothesis awaits further in-depth analysis at the genetic level and more exploration of the Rpf protein family in the future.

It was reported that D_2_O had no significant effect on spore germination and initial growth (Öberg et al., 2023; Zhang et al., 2020). Therefore, the method combining D_2_O isotope labeling with single-cell Raman spectroscopy was adopted to further verify the effect of RpfA protein on the metabolic activity during spore germination of *S. coelicolor* CGMCC 4.1658^T^, *S. niveus* JCM 4251^T^, *S. griseus* CGMCC 4.1419^T^, *S. laurentii* JCM 5063^T^, and *S. lusitanus* HBU208066^T^ (Figure 6). The experimental group with added RpfA protein had a higher cellular metabolic activity during spore germination compared to the control group, and the intensities of the C-D and C-H peaks in the experimental group were also higher, suggesting that due to the higher metabolic level, more intracellular substances were synthesized, resulting in a higher concentration of chemical bonds. The aforementioned studies fully demonstrate that the RpfA protein of *S. coelicolor* can promote the spore germination of its own as well as other *Streptomyces* species. Additionally, our previous work validated the activity of the Rpf protein from *Nocardiosis salophila* CGMCC 4.1195^T^, confirming its significant resuscitation effect on *N. salophila* in the viable but non-culturable (VBNC) state (Zhang et al., 2023). These findings have laid the foundation for establishing new methods to isolate unculturable or difficult-to-culture Actinomycetes based on the resuscitation function of Rpfs proteins.

A total of 264 strains (Supplementary Tables S3, S10) of phylum *Actinomycetotas* were isolated from six soil samples collected from the frozen soils of the Qinghai-Tibet Plateau. The result showed that the RpfA protein from *S. coelicolor* also promotes the isolation and cultivation of members of phylum *Actinomycetota*, with the most significant promotion effect observed on *Streptomyces* species, accounting for more than 50% of the total. There is a correlation between the quantity of strains of genus *Streptomyces* obtained and the concentration of RpfA protein added. In this study, the highest diversity and quantity of strains of genus *Streptomyces* were obtained when 10% RpfA protein was added. Based on the 16S rRNA gene sequences of these 137 *Streptomyces* strains, a phylogenetic tree was constructed (Figure 8). It was found that the five *Streptomyces* strains isolated from the control groups without RpfA protein addition clustered into two small groups, indicating that these species, which do not rely on the presence of RpfA protein for growth, have relatively close genetic relationships. This further suggests that the RpfA protein from *S. coelicolor* exhibits species selectivity in enhancing the cultivability of strains of genus *Streptomyces*, which is consistent with the previous observation that RpfA protein has differential effects on promoting spore germination activity among different *Streptomyces* species. The reason of why *S. liangshanensis* was only isolated from sample S3 and S6 without RpfA protein, but not from two samples with RpfA protein, might be that the species were not sensitive to the resuscitation effect of RpfA protein, or RpfA protein might have inhibitory effect on it to some extent. And different species of phylum *Actinomycetota* have varying sensitivities to Rpf protein. Most species of phylum *Actinomycetota* require higher concentrations of RpfA protein to promote their resuscitation and growth, while a few species only need low concentrations of RpfA protein to stimulate their growth, and high concentrations of RpfA protein may even be not conducive to their resuscitation growth. In general, the heterologously expressed RpfA protein from *S. coelicolor* can effectively enhance the cultivability of members of phylum *Actinomycetotas,* especially genus *Streptomyces*, of frozen soil samples on the Qinghai-Tibet Plateau.

The introduction of Rpf offers a novel approach for reviving difficult-to-culture microorganisms. Previous studies have also indicated that Rpf proteins derived from *M. luteus* and other species can enhance the cultivability of members of phylum *Actinomycetota* (Puspita et al., 2012; Wang et al., 2021). Furthermore, numerous studies have demonstrated that the addition of Rpfs proteins can resuscitate many strains of phylum *Actinomycetota* in a viable but non-culturable (VBNC) state that possess pollutant-degrading capabilities in the environment, thereby enhancing their biodegradation capacity for pollutants (Lothigius et al., 2010; Luo et al., 2019; Su et al., 2021; Su et al., 2018; Ye et al., 2020; Yu et al., 2020). However, there are few systematic studies on the effect of Rpf for cultivation of soil *Streptomyces*.

Among the isolates of phylum *Actinomycetota*, there were 21 potential new species, of which 8 belonged to genus *Streptomyces*, indicating that the RpfA protein of *S. coelicolor* could promote the isolation of new species of *Actinomycetota*, especially *Streptomyces*. The isolation rate of new species was also related to the type of medium used. Lopez et al. reported that Rpf proteins facilitated the isolation of 51 potential novel bacterial species (Lopez Marin et al., 2021). Wang et al. also isolated two new species of *Actinomycetota* from soil samples treated with Rpf (Wang et al., 2021). These studies provided a methodological reference for using Rpf proteins to excavate new species resources of phylum *Actinomycetota*.

## 5 Conclusion

Through this investigation, the purified RpfA protein of *S. coelicolor* was obtained by heterologous expression in *E. coli*. The RpfA protein of *S. coelicolor* significantly promotes the spore germination of itself and other *Streptomyces* species, and can significantly improve the cultivability of members of phylum *Actinomycetota*, especially genus *Streptomyces* and its potential new species in the frozen soil samples of the Qinghai-Tibet Plateau, the results support and verify the conclusion that RpfA protein can effectively increase the “cultivability” of actinobacteria under laboratory conditions. This provides a new method for exploring and preserving *Streptomyces* species resources in the unique environment of the Qinghai-Tibet Plateau and further developing new bioactive substances derived from *Streptomyces*.

In this study, the concentration of RpfA protein from *S. coelicolor* required to promote spore germination in itself and across species was observed to vary depending on the species. It is not the case that higher concentrations of RpfA protein leading to better spore germination promotion. This variation in activity and concentration may be related to differences in the genomes of various *Streptomyces* species, including the copy number, length, genetic structure of *rpf* genes (Tantivitayakul et al., 2020), host specificity, and differences in growth cycles (Flärdh and Buttner, 2009). For instance, *S. coelicolor* has five copies of *rpf* genes, while the other four *Streptomyces* strains tested have only four copies (Gong et al., 2022). In this study, RpfA protein was added at the early growth stage, leading to the speculation that different species exhibit varying sensitivities to RpfA protein at different growth stages. This is reflected in the significant differences in the concentration of RpfA protein required to promote spore germination in different *Streptomyces* species. This hypothesis awaits further analysis at the genetic level and more in-depth exploration of the Rpf protein family in the future.

## Data Availability

The datasets presented in this study can be found in online repositories. The names of the repository/repositories and accession number(s) can be found in the article/Supplementary material.

## Footnotes

^1^ https://www.uniprot.org/

^2^ https://www.ebi.ac.uk/Tools/hmmer/

^3^ https://www.ncbi.nlm.nih.gov

^4^ https://www.ezbiocloud.net/

^5^ https://itol.embl.de

^6^ https://www.expasy.org/

^7^ https://services.healthtech.dtu.dk/services/SignalP-5.0/

^8^ https://services.healthtech.dtu.dk/services/NetPhos-3.1/

^9^ https://www.ebi.ac.uk/Tools/hmmer/
