# Supplementary material for: Excavation of resources of Streptomyces species in frozen soils of the Qinghai-Tibet Plateau based on RpfA protein of Streptomyces coelicolor
Source: Front Microbiol. 2025 Apr 8;16:1557511. doi: 10.3389/fmicb.2025.1557511 (PMC12011840; doi:10.3389/fmicb.2025.1557511)
Supplement: Supplementary file 1 [file Data_Sheet_1.docx]

Supplementary Materials

Excavation of resources of *Streptomyces* species in frozen soils of the Qinghai-Tibet Plateau based on Rpf A protein of *S. coelicolor*

Yuxiao Xie^1†^, Jingjing Liu^1†^, Jun Ma^1^, Nan Shi^1,2,3^, Xiumin Zhang^1,2,3*^

^1^College of Life Sciences, Hebei University, Baoding 071002, Hebei Province, PR China.

^2^Key Laboratory of Microbial Diversity Research and Application of Hebei Province, Baoding 071002, Hebei Province, PR China.

^3^Engineering Research Center of Microbial Breeding and Conservation, Baoding 071002, Hebei Province, PR China.

^†^These authors contributed equally to this work.

*** Correspondence:**Corresponding Author: Xiumin Zhang
[zhxiumin1106@126.com](mailto:zhxiumin1106@126.com).

- **Supplementary Table 1.** Sixty type strains of *S**treptomyces* species associated with the determination of spore germination promoting activity of RpfA protein

| Number | Species | Accession numbers |
| --- | --- | --- |
| 1 | *S.* *ambofaciens* ATCC 23877^T^ | M27245 |
| 2 | *S.* *andamanensis* PCU 347^T^ | MT758159 |
| 3 | *S.* *anulatus* NBRC 13369^T^ | AB184875 |
| 4 | *S.* *ardesiacus* NBRC 15402^T^ | AB184653 |
| 5 | *S.* *atrovirens* NBRC 15388^T^ | AB184639 |
| 6 | *S.* *aureoverticillatus* NBRC 12742^T^ | AB249919 |
| 7 | *S.* *azureus* NRRL B-2655^T^ | EF178674 |

- **Supplementary Table 1.** Sixty type strains of *Streptomyces* species associated with the determination of spore germination promoting activity of RpfA protein (Continue)

| Number | Species | Accession numbers |
| --- | --- | --- |
| 8 | *S. cadmiisoli* ZFG47^T^ | MH300129 |
| 9 | *S. caelestis* NRRL 2418^T^ | X80824 |
| 10 | *S. coelicolor* NBRC 12854^T^ | AB184196 |
| 11 | *S. daghestanicus* JCM 4365^T^ | MT760521 |
| 12 | *S. flavofungini* NBRC 13371^T^ | AB184359 |
| 13 | *S. fulvorobeus* NBRC 15897^T^ | AB184711 |
| 14 | *S. fumanus* NBRC 13042^T^ | AB184273 |
| 15 | *S. gancidicus* NBRC 15412^T^ | AB184660 |
| 16 | *S. glaucescens* NBRC 12774^T^ | AB184843 |
| 17 | *S. gossypiisoli* TRM 44567^T^ | MN548415 |
| 18 | *S. griseus* DSM 40236^T^ | MK734067 |
| 19 | *S. halstedii* NRRL B-1238^T^ | EF178695 |
| 20 | *S. heliomycini* NBRC 15899^T^ | AB184712 |
| 21 | *S. hyderabadensis* OU 40^T^ | FM998652 |
| 22 | *S. hydrogenans* NBRC 13475^T^ | AB184868 |
| 23 | *S. kalpinensis* TRM 46509^T^ | KX021818 |
| 24 | *S. labedae* NBRC 15864^T^ | AB184704 |
| 25 | *S. laurentii* LMG 19959^T^ | AJ781342 |
| 26 | *S. longispororuber* NBRC 13488^T^ | AB184440 |
| 27 | *S. lusitanus* HBU208066^T^ | AB184424 |
| 28 | *S. macrolidinus* RY43-2^T^ | LC385744 |
| 29 | *S. malachitospinus* NBRC 101004^T^ | AB249954 |
| 30 | *S. marokkonensis* LMG 23016^T^ | AJ965470 |
| 31 | *S. microflavus* NBRC 13062^T^ | AB184284 |
| 32 | *S. niveus* NBRC 12804^T^ | AB184160 |

- **Supplementary Table 1.** Sixty type strains of *Streptomyces* species associated with the determination of spore germination promoting activity of RpfA protein (Continue)

| Number | Species | Accession numbers |
| --- | --- | --- |
| 33 | *S. nogalater* JCM 4799^T^ | AB045886 |
| 34 | *S. nymphaeiformis* SFB5A^T^ | MH392705 |
| 35 | *S. omiyaensis* NRRL B-1587^T^ | EF178697 |
| 36 | *S. pseudogriseolus* NBRC 12902^T^ | AB184232 |
| 37 | *S. purpurascens* JCM 4509^T^ | AB045888 |
| 38 | *S. purpureus* NBRC 13927^T^ | AB184547 |
| 39 | *S. speibonae* PK-Blue^T^ | AF452714 |
| 40 | *S. spinoverrucosus* NBRC 14228^T^ | AB184578 |
| 41 | *S. tacrolimicus* ATCC 55098^T^ | AB217601 |
| 42 | *S. tendae* ATCC 19812^T^ | D63873 |
| 43 | *S. termitum* NBRC 13087^T^ | AB184302 |
| 44 | *S. thermocarboxydus* NBRC 16323^T^ | AB249926 |
| 45 | *S. thermospinosisporus* AT10^T^ | AF333113 |
| 46 | *S. thermovulgaris* DSM 40444^T^ | Z68094 |
| 47 | *S. thinghirensis* S10^T^ | FM202482 |
| 48 | *S. tritici* NEAU-A4^T^ | KY744943 |
| 49 | *S. tritolerans* DAS 165^T^ | DQ345779 |
| 50 | *S. tuirus* NBRC 15617^T^ | AB184690 |
| 51 | *S. tunisiensis* CN 207^T^ | KF697135 |
| 52 | *S. ureilyticus* YC419^T^ | KU323816 |
| 53 | *S. variegatus* NBRC 15462^T^ | AB184688 |
| 54 | *S. venetus* CMU-AB225^T^ | LC073310 |
| 55 | *S. venezuelae* JCM 4526^T^ | AB045890 |
| 56 | *S. vietnamensis* GIMV4.0001^T^ | DQ311081 |
| 57 | *S. violaceochromogenes* NBRC 13100^T^ | AB184312 |
| 58 | *S. violaceorectus* NBRC 13102^T^ | AB184314 |

- **Supplementary Table 1.** Sixty type strains of *Streptomyces* species associated with the determination of spore germination promoting activity of RpfA protein (Continue)

| Number | Species | Accession numbers |
| --- | --- | --- |
| 59 | *S. violaceoruber* DSM 40049^T^ | AF503492 |
| 60 | *S. violaceorubidus* LMG 20319^T^ | AJ781374 |

- **Supplementary Table 2.** Spore germination rates of *Streptomyces* species at different culture times with the addition of different concentrations of Rpf protein

| Species | CRpfA^*^ | Spore germination rates (%) | | | | | |
| --- | --- | --- | --- | --- | --- | --- | --- |
|  |  | 12h | 24h | 36h | 48h | 60h | 72h |
| *S. coelicolor* | 0% | 0 | 0 | 20.3 | 34.2 | 34.2 | 34.2 |
|  | 0.1% | 0 | 0 | 15.3 | 20.3 | 20.3 | 20.3 |
|  | 1% | 0 | 0 | 16.8 | 20.3 | 20.3 | 20.3 |
|  | 10% | 64.9 | 76.7 | 76.7 | 79.2 | 79.2 | 79.2 |
| *S. niveus* | 0% | 0 | 25 | 29.2 | 29.2 | 29.2 | 29.2 |
|  | 0.1% | 0 | 37.5 | 50 | 58.3 | 58.3 | 58.3 |
|  | 1% | 0 | 16.7 | 50 | 50 | 50 | 50 |
|  | 10% | 0 | 62.5 | 79.2 | 79.2 | 83.3 | 83.3 |
| *S. griseus* | 0% | 0 | 8.3 | 44.4 | 47.2 | 47.2 | 47.2 |
|  | 0.1% | 0 | 2.8 | 30.6 | 30.6 | 33.3 | 33.3 |
|  | 1% | 0 | 8.3 | 61.1 | 63.9 | 69.4 | 69.4 |
|  | 10% | 0 | 5.6 | 47.2 | 52.8 | 52.8 | 52.8 |
| *S. laurentii* | 0% | 0 | 4.7 | 9.4 | 9.4 | 9.4 | 9.4 |
|  | 0.1% | 0 | 65.6 | 73.4 | 73.4 | 73.4 | 73.4 |
|  | 1% | 0 | 4.7 | 9.4 | 9.4 | 9.4 | 9.4 |
|  | 10% | 0 | 1.6 | 3.1 | 3.1 | 3.1 | 3.1 |
| *S.* *lusitanus* | 0% | 0 | 0 | 18.6 | 22.9 | 22.9 | 22.9 |
|  | 0.1% | 0 | 0 | 68.6 | 71.8 | 71.8 | 71.8 |
|  | 1% | 0 | 0 | 17.6 | 20.7 | 20.7 | 20.7 |
|  | 10% | 0 | 0 | 18.6 | 23.9 | 23.9 | 23.9 |

*CRpfA represents the concentration of RpfA protein.

- **Supplementary Table 3.** Similarities of 16S rRNA genes between the *Streptomyces* strains isolated from different samples and theirs closest type strains

| CRpf(%)^*^ | Samples | Culture media | Strain numbers | Accession numbers | Most similar strains | Similarities (%) |
| --- | --- | --- | --- | --- | --- | --- |
| 10 | S1 | R_2_A | QTP241047 | PP837102 | *S. turgidiscabies* ATCC 700248^T^ | 99.30 |
|  |  | R_2_A | QTP241011 | PP837066 | *S. microflavus* NRRL B-2156^T^ | 99.30 |
|  |  | R_2_A | QTP241006 | PP837061 | *S. bobili* NBRC 16166^T^ | 99.44 |
|  |  | R_2_A | QTP241007 | PP837062 | *S. olivoviridis* NBRC 12897^T^ | 99.51 |
|  |  | R_2_A | QTP241080 | PP837135 | *S. olivoviridis* NBRC 12897^T^ | 99.65 |
|  |  | R_2_A | QTP241059 | PP837113 | *S. olivoviridis* NBRC 12897^T^ | 99.86 |
|  |  | R_2_A | QTP241070 | PP837125 | *S. caniscabiei* NE06-02D^T^ | 99.16 |
|  |  | R_2_A | QTP241127 | PP837204 | *S. finlayi* NBRC 13201^T^ | 99.51 |
|  |  | R_2_A | QTP241096 | PP837151 | *S. finlayi* CSSP541^T^ | 99.72 |
|  |  | R_2_A | QTP241104 | PP837194 | *S. scopiformis* A25^T^ | 99.65 |
|  |  | R_2_A | QTP241125 | PP837213 | *S. finlayi* NBRC 13201^T^ | 99.58 |
|  |  | glucose-tryptone | QTP241057 | PP837111 | *S. tateyamensis* Sp080513SC-30^T^ | 97.82 |
|  |  | glucose-tryptone | QTP241058 | PP837112 | *S. finlayi* NBRC 13201^T^ | 99.72 |
|  |  | glucose-tryptone | QTP241060 | PP837114 | *S. olivoviridis* NBRC 12897^T^ | 99.51 |
|  |  | glucose-tryptone | QTP241068 | PP837123 | *S. finlayi* NBRC 13201^T^ | 99.65 |
|  |  | glucose-tryptone | QTP241078 | PP837133 | *S. bobili* NBRC 16166^T^ | 99.58 |
|  |  | glucose-tryptone | QTP241074 | PP837129 | *S. finlayi* CSSP541^T^ | 99.65 |
|  |  | glucose-tryptone | QTP241128 | PP837223 | *S. graminofaciens* CSSP419^T^ | 99.58 |
|  |  | glucose-tryptone | QTP241082 | PP837137 | *S. cyaneofuscatus* CSSP436^T^ | 99.51 |
|  | S2 | R_2_A | QTP242038 | PP837092 | *S. pratensis* ch24^T^ | 99.72 |

*CRpfA represents the concentration of RpfA protein.

- **Supplementary Table 3.** Similarities of 16S rRNA genes between the *Streptomyces* strains isolated from different samples and theirs closest type strains (Continue)

| CRpf(%)^*^ | Samples | Culture media | Strain numbers | Accession numbers | Most similar strains | Similarities (%) |
| --- | --- | --- | --- | --- | --- | --- |
| 10 | S2 | R_2_A | QTP242008 | PP837063 | *S. turgidiscabies* ATCC 700248^T^ | 99.23 |
|  |  | R_2_A | QTP242009 | PP837064 | *S. badius* CSSP536^T^ | 99.65 |
|  |  | R_2_A | QTP242010 | PP837065 | *S. badius* CSSP536^T^ | 99.58 |
|  |  | R_2_A | QTP242012 | PP837067 | *S. turgidiscabies* ATCC 700248^T^ | 99.30 |
|  |  | R_2_A | QTP242014 | PP837069 | *S. microflavus* NBRC 13062^T^ | 99.72 |
|  |  | R_2_A | QTP242016 | PP837071 | *S. olivoviridis* NBRC 12897^T^ | 99.86 |
|  |  | R_2_A | QTP242017 | PP837072 | *S. cyaneofuscatus* CSSP436^T^ | 99.44 |
|  |  | R_2_A | QTP242018 | PP837073 | *S. chlorus* BK125^T^ | 98.52 |
|  |  | R_2_A | QTP242019 | PP837074 | *S. novaecaesareae* NBRC 13368^T^ | 99.30 |
|  |  | R_2_A | QTP242020 | PP837075 | *S. microflavus* NBRC 13062^T^ | 99.72 |
|  |  | R_2_A | QTP242135 | PP837171 | *S. clavifer* NBRC 15398^T^ | 99.65 |
|  |  | R_2_A | QTP242136 | PP837172 | *S. turgidiscabies* ATCC 700248^T^ | 99.37 |
|  |  | R_2_A | QTP242055 | PP837109 | *S. cahuitamycinicus* 13K301^T^ | 98.24 |
|  |  | R_2_A | QTP242081 | PP837136 | *S. pratensis* ch24^T^ | 99.72 |
|  |  | R_2_A | QTP242075 | PP837130 | *S. praecox* CSSP720^T^ | 99.79 |
|  |  | R_2_A | QTP242095 | PP837150 | *S. cahuitamycinicus* 13K301^T^ | 98.39 |
|  |  | R_2_A | QTP242107 | PP837161 | *S. cahuitamycinicus* 13K301^T^ | 98.25 |
|  |  | R_2_A | QTP242108 | PP837162 | *S. europaeiscabiei* CFBP 4497^T^ | 99.58 |

*CRpfA represents the concentration of RpfA protein.

- **Supplementary Table 3.** Similarities of 16S rRNA genes between the *Streptomyces* strains isolated from different samples and theirs closest type strains (Continue)

| CRpf(%)^*^ | Samples | Culture media | Strain numbers | Accession numbers | Most similar strains | Similarities (%) |
| --- | --- | --- | --- | --- | --- | --- |
| 10 | S2 | R_2_A | QTP242109 | PP837221 | *S. olivoviridis* NBRC 12897^T^ | 98.95 |
|  |  | R_2_A | QTP242110 | PP837163 | *S. cyaneofuscatus* CSSP436^T^ | 99.58 |
|  |  | R_2_A | QTP242111 | PP837164 | *S. cahuitamycinicus* 13K301^T^ | 98.38 |
|  |  | glucose-tryptone | QTP242039 | PP837093 | *S. praecox* CSSP720^T^ | 99.65 |
|  |  | glucose-tryptone | QTP242130 | PP837203 | *S. turgidiscabies* ATCC 700248^T^ | 99.23 |
|  |  | glucose-tryptone | QTP242088 | PP837143 | *S. pratensis* ch24^T^ | 99.16 |
|  |  | glucose-tryptone | QTP242101 | PP837157 | *S. cyaneofuscatus* CSSP436^T^ | 99.15 |
|  | S3 | R_2_A | QTP243062 | PP837116 | *S. praecox* CSSP720^T^ | 99.79 |
|  |  | R_2_A | QTP243065 | PP837120 | *S. novaecaesareae* NBRC 13368^T^ | 99.23 |
|  |  | R_2_A | QTP243066 | PP837121 | *S. caniscabiei* NE06-02D^T^ | 99.23 |
|  |  | R_2_A | QTP243067 | PP837122 | *S. camponoticapitis* 2H-TWYE14^T^ | 99.29 |
|  |  | R_2_A | QTP243072 | PP837127 | *S. caniscabiei* NE06-02D^T^ | 99.58 |
|  |  | R_2_A | QTP243120 | PP837187 | *S. cyaneofuscatus* NBRC 13190^T^ | 99.50 |
|  |  | R_2_A | QTP243077 | PP837132 | *S. badius* CSSP536^T^ | 99.72 |
|  |  | glucose-tryptone | QTP243028 | PP837083 | *S. microflavus* NRRL B-2156^T^ | 99.51 |
|  |  | glucose-tryptone | QTP243092 | PP837147 | *S. pratensis*ch ch24^T^ | 99.86 |
|  |  | glucose-tryptone | QTP243102 | PP837158 | *S. pratensis*ch ch24^T^ | 99.58 |
|  |  | glucose-tryptone | QTP243123 | PP837178 | *S. dioscori* A217^T^ | 99.09 |

*CRpfA represents the concentration of RpfA protein.

- **Supplementary Table 3.** Similarities of 16S rRNA genes between the *Streptomyces* strains isolated from different samples and theirs closest type strains (Continue)

| CRpf(%)^*^ | Samples | Culture media | Strain numbers | Accession numbers | Most similar strains | Similarities (%) |
| --- | --- | --- | --- | --- | --- | --- |
| 10 | S3 | glucose-tryptone | QTP243029 | PP837084 | *S*．*novaecaesareae* NBRC13368^T^ | 99.09 |
|  |  | glucose-tryptone | QTP243033 | PP837228 | *S*．*olivoviridis* NBRC 12897^T^ | 99.58 |
|  |  | glucose-tryptone | QTP243053 | PP837176 | *S*．*microflavus* NRRL B-2156^T^ | 100.00 |
|  |  | glucose-tryptone | QTP243054 | PP837108 | *S. pratensis* ch24^T^ | 99.72 |
|  |  | glucose-tryptone | QTP243040 | PP837095 | *S*．*cyaneofuscatus* CSSP436^T^ | 98.66 |
|  | S4 | R_2_A | QTP245002 | PP837057 | *S*．*chryseus* NBRC 13377^T^ | 99.72 |
|  |  | R_2_A | QTP245003 | PP837058 | *S*．*liliiviolaceus* BH-SS-21^T^ | 98.94 |
|  |  | R_2_A | QTP245005 | PP837060 | *S*．*avidinii* NRRL 3077^T^ | 99.44 |
|  |  | R_2_A | QTP245021 | PP837076 | *S*．*avidinii* NRRL 3077^T^ | 99.51 |
|  |  | R_2_A | QTP245023 | PP837078 | *S*．*gelidistatuariae* ISLP-3^T^ | 99.28 |
|  |  | R_2_A | QTP245026 | PP837081 | *S*．*lacrimifluminis* Z1027^T^ | 98.31 |
|  |  | R_2_A | QTP245027 | PP837082 | *S. caniscabiei* NE06-02D^T^ | 99.23 |
|  | S5 | R_2_A | QTP244001 | PP837056 | *S. cyaneofuscatus* CSSP436^T^ | 99.72 |
|  |  | R_2_A | QTP244076 | PP837131 | *S*．*bobili* NBRC 16166^T^ | 99.37 |
|  |  | R_2_A | QTP244124 | PP837211 | *S*．*europaeiscabiei* CFBP 4497^T^ | 99.65 |
|  |  | R_2_A | QTP244087 | PP837142 | *S*．*microflavus* NRRL B-2156^T^ | 99.65 |
|  |  | R_2_A | QTP244084 | PP837139 | *S*．*europaeiscabiei* CFBP 4497^T^ | 99.79 |
|  |  | glucose-tryptone | QTP244091 | PP837146 | *S*．*microflavus* NBRC 13062^T^ | 99.65 |
|  |  | glucose-tryptone | QTP244100 | PP837156 | *S*．*liangshanensis* QMT-12^T^ | 99.50 |

*CRpfA represents the concentration of RpfA protein.

- **Supplementary Table 3.** Similarities of 16S rRNA genes between the *Streptomyces* strains isolated from different samples and theirs closest type strains (Continue)

| CRpf(%)^*^ | Samples | Culture media | Strain numbers | Accession numbers | Most similar strains | Similarities (%) |
| --- | --- | --- | --- | --- | --- | --- |
| 10 | S5 | glucose-tryptone | QTP244132 | PP837168 | *S. nojiriensis* NBRC 13794^T^ | 99.65 |
|  |  | glucose-tryptone | QTP244133 | PP837169 | *S. microflavus* DSM 40593^T^ | 99.65 |
|  |  | glucose-tryptone | QTP244083 | PP837138 | *S. chryseus* NBRC 13377^T^ | 99.79 |
|  | S6 | R_2_A | QTP246118 | PP837185 | *S. pratens* BK138^T^ | 99.44 |
|  |  | glucose-tryptone | QTP246119 | PP837177 | *S. urticae* NEAU-PCY-1^T^ | 98.73 |
| 1 | S1 | R_2_A | QTP241175 | PP837313 | *S. tateyamensis* Sp080513SC-30^T^ | 98.07 |
|  |  | R_2_A | QTP241186 | PP837189 | *S. finlayi* NBRC 13201^T^ | 99.51 |
|  |  | glucose-tryptone | QTP241159 | PP837247 | *S. praecox* CSSP720^T^ | 99.79 |
|  |  | glucose-tryptone | QTP241158 | PP837246 | *S. pratensis* ch24^T^ | 99.79 |
|  | S2 | R_2_A | QTP242142 | PP837231 | *S. praecox* CSSP720^T^ | 99.65 |
|  |  | R_2_A | QTP242151 | PP837239 | *S. turgidiscabies* NBRC 16080^T^ | 99.23 |
|  |  | R_2_A | QTP242192 | PP837225 | *S. praecox* CSSP720^T^ | 99.79 |
|  |  | R_2_A | QTP242168 | PP837256 | *S. badius* CSSP536^T^ | 99.44 |
|  |  | glucose-tryptone | QTP242189 | PP837264 | *S. turgidiscabies* ATCC 700248^T^ | 99.23 |
|  | S3 | R_2_A | QTP243157 | PP837245 | *S. cyaneofuscatus* CSSP436^T^ | 99.37 |
|  |  | R_2_A | QTP243160 | PP837248 | *S. microflavus* DSM 40593^T^ | 99.79 |
|  |  | R_2_A | QTP243180 | PP837192 | *S. camponoticapitis* 2H-TWYE14^T^ | 99.22 |
|  |  | R_2_A | QTP243181 | PP837180 | *S. lacrimifluminis* Z1027^T^ | 98.67 |
|  |  | glucose-tryptone | QTP243154 | PP837242 | *S. olivoviridis* NBRC 12897^T^ | 99.86 |

*CRpfA represents the concentration of RpfA protein.

- **Supplementary Table 3.** Similarities of 16S rRNA genes between the *Streptomyces* strains isolated from different samples and theirs closest type strains (Continue)

| CRpf(%)^*^ | Samples | Culture media | Strain numbers | Accession numbers | Most similar strains | Similarities (%) |
| --- | --- | --- | --- | --- | --- | --- |
| 1 | S3 | glucose-tryptone | QTP243162 | PP837250 | *S. caniscabiei* NE06-02D^T^ | 99.44 |
|  |  | glucose-tryptone | QTP243163 | PP837251 | *S. microflavus* NRRL B-2156^T^ | 99.79 |
|  |  | glucose-tryptone | QTP243172 | PP837260 | *S. novaecaesareae* NBRC 13368^T^ | 99.29 |
|  | S4 | R_2_A | QTP245174 | PP837262 | *S. microflavus* NRRL B-2156^T^ | 99.72 |
|  |  | R_2_A | QTP245146 | PP837235 | *S. avidinii* NRRL3077^T^ | 99.65 |
|  |  | R_2_A | QTP245155 | PP837243 | *S. brevispora* BK160^T^ | 99.65 |
|  |  | R_2_A | QTP245141 | PP837230 | *S. chryseus* NBRC 13377^T^ | 99.65 |
|  |  | glucose-tryptone | QTP245187 | PP837224 | *S. microflavus* NBRC 13062^T^ | 99.51 |
|  | S5 | R_2_A | QTP244183 | PP837226 | *S. olivoviridis* NBRC 12897^T^ | 99.58 |
|  |  | glucose-tryptone | QTP244190 | PP837265 | *S. chryseus* NBRC 13377^T^ | 99.86 |
|  |  | glucose-tryptone | QTP244140 | PP837229 | *S. liangshanensis* QMT-12^T^ | 99.50 |
|  | S6 | R_2_A | QTP246176 | PP837198 | *S. lavendulae* NBRC 12789^T^ | 99.78 |
|  |  | R_2_A | QTP246177 | PP837181 | *S. urticae* NEAU-PCY-1^T^ | 98.32 |
| 0.1 | S1 | R_2_A | QTP241229 | PP837210 | *S.pratensis* ch24^T^ | 99.79 |
|  |  | R_2_A | QTP241230 | PP837218 | *S. sannanensis* NBRC 14239^T^ | 98.87 |
|  |  | R_2_A | QTP241231 | PP837208 | *S. olivoviridis* NBRC 12897^T^ | 99.58 |
|  |  | R_2_A | QTP241217 | PP837284 | *S. finlayi* NBRC 13201^T^ | 99.65 |
|  |  | R_2_A | QTP241208 | PP837275 | *S. microflavus* NRRL B-2156^T^ | 99.72 |
|  |  | R_2_A | QTP241209 | PP837276 | *S. olivoviridis* NBRC 12897^T^ | 99.72 |

*CRpfA represents the concentration of RpfA protein.

- **Supplementary Table 3.** Similarities of 16S rRNA genes between the *Streptomyces* strains isolated from different samples and theirs closest type strains (Continue)

| CRpf(%)^*^ | Samples | Culture media | Strain numbers | Accession numbers | Most similar strains | Similarities (%) |
| --- | --- | --- | --- | --- | --- | --- |
| 0.1 | S1 | glucose-tryptone | QTP241216 | PP837283 | *S. finlayi* CSSP541^T^ | 99.65 |
|  | S2 | R_2_A | QTP242233 | PP837205 | *S. praecox* CSSP720^T^ | 99.79 |
|  |  | R_2_A | QTP242237 | PP837219 | *S. turgidiscabies* ATCC 700248^T^ | 99.02 |
|  |  | glucose-tryptone | QTP242213 | PP837280 | *S. badius* CSSP536^T^ | 99.58 |
|  |  | glucose-tryptone | QTP242214 | PP837281 | *S. praecox* CSSP720^T^ | 99.86 |
|  | S3 | R_2_A | QTP243204 | PP837272 | *S. cyaneofuscatus* NBRC 13190^T^ | 99.36 |
|  |  | R_2_A | QTP243205 | PP837119 | *S. cyaneofuscatus* CSSP436^T^ | 99.72 |
|  |  | glucose-tryptone | QTP243223 | PP837287 | *S. novaecaesareae* NBRC 13368^T^ | 99.09 |
|  |  | glucose-tryptone | QTP243224 | PP837288 | *S. microflavus* NBRC 13062^T^ | 99.79 |
|  |  | R_2_A | QTP243207 | PP837274 | *S. caniscabiei* NE06-02D^T^ | 99.30 |
|  |  | glucose-tryptone | QTP243215 | PP837282 | *S. clavifer* NBRC 15398^T^ | 99.65 |
|  |  | glucose-tryptone | QTP243219 | PP837193 | *S. olivoviridis* NBRC 12897^T^ | 99.44 |
|  | S4 | R_2_A | QTP245202 | PP837315 | *S. avidinii* NRRL 3077^T^ | 99.58 |
|  |  | R_2_A | QTP245198 | PP837270 | *S. lacrimifluminis* Z1027^T^ | 98.53 |
|  |  | R_2_A | QTP245203 | PP837316 | *S. clavifer* NBRC 15398^T^ | 99.86 |
|  | S5 | R_2_A | QTP245232 | PP837214 | *S. cyaneofuscatus* CSSP436^T^ | 99.23 |
|  |  | glucose-tryptone | QTP244235 | PP837291 | *S. spororaveus* NBRC 15456^T^ | 99.71 |
|  |  | glucose-tryptone | QTP244236 | PP837222 | *S. nojiriensis* NBRC 13794^T^ | 99.37 |
|  |  | glucose-tryptone | QTP244212 | PP837279 | *S. microflavus* NBRC 13062^T^ | 99.72 |

*CRpfA represents the concentration of RpfA protein.

- **Supplementary Table 3.** Similarities of 16S rRNA genes between the *Streptomyces* strains isolated from different samples and theirs closest type strains (Continue)

| CRpf(%)^*^ | Samples | Culture media | Strain numbers | Accession numbers | Most similar strains | Similarities (%) |
| --- | --- | --- | --- | --- | --- | --- |
| 0 | S1 | glucose-tryptone | QTP241248 | PP837297 | *S. tateyamensis* Sp080513SC-30^T^ | 97.82 |
|  | S3 | R_2_A | QTP243249 | PP837298 | *S. olivoviridis* NBRC 12897^T^ | 98.95 |
|  |  | glucose-tryptone | QTP243252 | PP837301 | *S. liangshanensis* QMT-12^T^ | 99.64 |
|  | S5 | R_2_A | QTP244251 | PP837300 | *S. cyaneofuscatus* CSSP436^T^ | 99.30 |
|  | S6 | R_2_A | QTP246246 | PP837174 | *S. liangshanensis* QMT-12^T^ | 99.42 |

*CRpfA represents the concentration of RpfA protein.

- **Supplementary Table 4.** Statistics of *Streptomyces* species and quantities isolated from sample S1 adding different the concentrations of RpfA protein

| Species | Number of strains | | | | |
| --- | --- | --- | --- | --- | --- |
|  | CRpfA 0% | CRpfA 0.1% | CRpfA 1% | CRpfA 10% |  |
| *S．turgidiscabies* |  |  |  | 1 |  |
| *S．microflavus* |  | 1 |  | 1 |  |
| *S．bobili* |  |  |  | 2 |  |
| *S．olivoviridis* |  | 2 |  | 4 |  |
| *S．finlayi* |  | 2 | 1 | 6 |  |
| *S．scopiformis* |  |  |  | 1 |  |
| *S．tateyamensis* | 1 |  | 1 | 1 |  |
| *S．graminofaciens* |  |  |  | 1 |  |
| *S．cyaneofuscatus* |  |  |  | 1 |  |
| *S．pratensis* |  | 1 | 1 |  |  |
| *S．praecox* |  |  | 1 |  |  |
| *S．caniscabiei* |  |  |  | 1 |  |
| *S．sannanensis* |  | 1 |  |  |  |
| Total | 1 | 7 | 4 | 19 |  |

CRpfA represents the concentration of RpfA protein.

- **Supplementary Table 5.** Statistics of *Streptomyces* species and quantities isolated from sample S2 adding different the concentrations of RpfA protein

| Species | Number of strains | | | |
| --- | --- | --- | --- | --- |
|  | CRpfA 0% | CRpfA 0.1% | CRpfA 1% | CRpfA 10% |
| *S．turgidiscabies* |  | 1 | 2 | 4 |
| *S．microflavus* |  |  |  | 2 |
| *S．olivoviridis* |  |  |  | 2 |
| *S．cyaneofuscatus* |  |  |  | 3 |
| *S．pratensis* |  |  |  | 3 |
| *S．badius* |  | 1 | 1 | 2 |
| *S．chlorus* |  |  |  | 1 |
| *S．novaecaesareae* |  |  |  | 1 |
| *S．clavifer* |  |  |  | 1 |
| *S．cahuitamycinicus* |  |  |  | 4 |
| *S．praecox* |  | 2 | 2 | 2 |
| *S．europaeiscabiei* |  |  |  | 1 |
| Total | 0 | 4 | 5 | 26 |

CRpfA represents the concentration of RpfA protein.

- **Supplementary Table 6.** Statistics of *Streptomyces* species and quantities isolated from sample S3 adding different the concentrations of RpfA protein

| Species | Number of strains | | | |
| --- | --- | --- | --- | --- |
|  | CRpfA 0% | CRpfA 0.1% | CRpfA 1% | CRpfA 10% |
| *S．microflavus* |  | 1 | 2 | 2 |
| *S．olivoviridis* | 1 | 1 | 1 | 1 |
| *S．cyaneofuscatus* |  | 2 | 1 | 2 |
| *S．pratensis* |  |  |  | 3 |
| *S．badius* |  |  |  | 1 |
| *S．novaecaesareae* |  | 1 | 1 | 2 |
| *S．clavifer* |  | 1 |  |  |
| *S．praecox* |  |  |  | 1 |
| *S．caniscabiei* |  | 1 | 1 | 2 |
| *S．camponoticapitis* |  |  | 1 | 1 |
| *S．dioscori* |  |  |  | 1 |
| *S．lacrimifluminis* |  |  | 1 |  |
| *S．caniscabiei* |  |  |  |  |
| *S．liangshanensis* | 1 |  |  |  |
| Total | 2 | 7 | 8 | 16 |

CRpfA represents the concentration of RpfA protein.

- **Supplementary Table 7.** Statistics of *Streptomyces* species and quantities isolated from sample S4 adding different the concentrations of RpfA protein

| Species | Numbers of strains | | | |
| --- | --- | --- | --- | --- |
|  | CRpfA 0% | CRpfA 0.1% | CRpfA 1% | CRpfA 10% |
| *S．microflavus* |  | 1 | 2 |  |
| *S. avidinii* |  |  | 1 | 2 |
| *S. brevispora* |  |  | 1 |  |
| *S*. *lacrimifluminis* |  |  |  | 1 |
| *S．cyaneofuscatus* |  | 1 |  |  |
| *S. caniscabiei* |  |  |  | 1 |
| *S*．*liliiviolaceus* |  |  |  | 1 |
| *S．chryseus* |  |  | 1 | 1 |
| *S*．*gelidistatuariae* |  |  |  | 1 |
| *S．nojiriensis* |  | 1 |  |  |
| *S．spororaveus* |  | 1 |  |  |
| Total | 0 | 4 | 5 | 7 |

CRpfA represents the concentration of RpfA protein.

- **Supplementary Table 8.** Statistics of *Streptomyces* species and quantities isolated from sample S5 adding different the concentrations of RpfA protein

| Species | Number of strains | | | |
| --- | --- | --- | --- | --- |
|  | CRpfA 0% | CRpfA 0.1% | CRpfA 1% | CRpfA 10% |
| *S．olivoviridis* |  |  | 1 |  |
| *S．cyaneofuscatus* | 1 |  |  | 1 |
| *S*．*bobili* |  |  |  | 1 |
| *S*．*europaeiscabiei* |  |  |  | 2 |
| *S*．*microflavus* |  |  |  | 3 |
| *S．clavifer* |  | 1 |  |  |
| *S．chryseus* |  |  | 1 | 1 |
| *S. liangshanensis* |  |  | 1 | 1 |
| *S．avidinii* |  | 1 |  |  |
| *S．lacrimifluminis* |  | 1 |  |  |
| *S. nojiriensis* |  |  |  | 1 |
| Total | 1 | 3 | 3 | 10 |

CRpfA represents the concentration of RpfA protein.

- **Supplementary Table 9.** Statistics of *Streptomyces* species and quantities isolated from sample S6 adding different the concentrations of RpfA protein

| Species | Numbers of strains | | | |
| --- | --- | --- | --- | --- |
|  | CRpfA 0% | CRpfA 0.1% | CRpfA 1% | CRpfA 10% |
| *S．pratensis* |  |  |  | 1 |
| *S．liangshanensis* | 1 |  |  |  |
| *S．urticae* |  |  | 1 | 1 |
| *S．lavendulae* |  |  | 1 |  |
| Total | 1 | 0 | 2 | 2 |

CRpfA represents the concentration of RpfA protein.

- **Supplementary Table 10.** Similarities of 16S rRNA genes between the non-*Streptomyces* strains of *Actinomycetota* isolated from different samples and their most similar type strains

| CRpf(%)^*^ | Samples | Culture media | Strain numbers | Accession numbers | Most similar strains | Similarities (%) |
| --- | --- | --- | --- | --- | --- | --- |
| 10 | S1 | R_2_A | QTP241049 | PP837104 | *Oerskovia turbata* NBRC 15015^T^ | 99.23 |
|  |  | R_2_A | QTP241043 | PP837098 | *Promicromonospora alba* 1C-HV12^T^ | 99.44 |
|  |  | R_2_A | QTP241031 | PP837086 | *Oerskovia enterophila* DSM 43852^T^ | 99.22 |
|  |  | R_2_A | QTP241032 | PP837087 | *Embleya scabrispora* NBRC 100760^T^ | 99.44 |
|  |  | R_2_A | QTP241024 | PP837079 | *Cellulomonas xylanilytica* XIL11^T^ | 98.52 |
|  |  | R_2_A | QTP241025 | PP837080 | *Cellulomonas timonensis* SN7^T^ | 99.79 |
|  |  | R_2_A | QTP241015 | PP837070 | *Cellulomonas humilata* ATCC 25174^T^ | 98.72 |
|  |  | R_2_A | QTP241056 | PP837110 | *Isoptericola variabilis* MX5^T^ | 99.21 |
|  |  | R_2_A | QTP241085 | PP837140 | *Micromonospora saelicesensis* Lupac 09^T^ | 99.50 |
|  |  | R_2_A | QTP241086 | PP837141 | *Micromonospora zeae* NEAU-gq9^T^ | 99.29 |
|  |  | R_2_A | QTP241073 | PP837128 | *Nocardia fluminea* DSM 44489^T^ | 99.15 |
|  |  | R_2_A | QTP241105 | PP837160 | *Nocardia fluminea* DSM 44489^T^ | 99.36 |
|  |  | R_2_A | QTP241106 | PP837195 | *Isoptericola variabilis* MX5^T^ | 99.01 |
|  |  | R_2_A | QTP241112 | PP837165 | *Nocardia fluminea* DSM 44489^T^ | 99.15 |
|  |  | R_2_A | QTP241113 | PP837166 | *Cellulomonas xylanilytica* XIL11^T^ | 99.08 |
|  |  | glucose-tryptone | QTP241064 | PP837118 | *Nocardia fluminea* DSM 44489^T^ | 99.37 |
|  |  | glucose-tryptone | QTP241069 | PP837124 | *Oerskovia turbata* 27^T^ | 99.51 |
|  |  | glucose-tryptone | QTP241061 | PP837115 | *Nocardia goodfellowii* A2012^T^ | 99.29 |
|  |  | glucose-tryptone | QTP241052 | PP837107 | *Nocardia goodfellowii* A2012^T^ | 99.29 |
|  | S2 | R_2_A | QTP242036 | PP837090 | *Nocardia salmonicida* DSM 4490^T^ | 99.79 |
|  |  | R_2_A | QTP242037 | PP837091 | *Cellulomonas xylanilytica* XIL11^T^ | 99.01 |

*CRpfA represents the concentration of RpfA protein.

**Supplementary Table 10.** Similarities of 16S rRNA genes between the non-*Streptomyces* strains of *Actinomycetota* isolated from different samples and their most similar type strains (Continue)

| CRpf(%)^*^ | Samples | Culture media | Strain numbers | Accession numbers | Most similar strains | Similarities (%) |
| --- | --- | --- | --- | --- | --- | --- |
| 10 | S2 | R_2_A | QTP242048 | PP837103 | *Isoptericola* *variabilis* MX5^T^ | 99.08 |
|  |  | R_2_A | QTP242013 | PP837068 | *Nocardia* *fluminea* DSM 44489^T^ | 99.15 |
|  |  | R_2_A | QTP242044 | PP837099 | *Nocardia* *salmonicida* R89^T^ | 99.64 |
|  |  | R_2_A | QTP242137 | PP837173 | *Nonomuraea glycinis* NEAU BB2C19^T^ | 99.44 |
|  |  | R_2_A | QTP242094 | PP837149 | *Micromonospora lycii* NEAU gq11^T^ | 99.50 |
|  |  | R_2_A | QTP242114 | PP837167 | *Nocardia salmonicida* DSM 4490^T^ | 99.50 |
|  |  | R_2_A | QTP242131 | PP837206 | *Micromonospora zeae* NEAU gq9^T^ | 99.57 |
|  |  | R_2_A | QTP242126 | PP837209 | *Nocardia salmonicida* DSM 4490^T^ | 99.51 |
|  |  | glucose-tryptone | QTP242093 | PP837148 | *Oerskovia turbata* 27^T^ | 99.86 |
|  |  | glucose-tryptone | QTP242103 | PP837159 | *Nocardia salmonicida* R89^T^ | 99.58 |
|  |  | glucose-tryptone | QTP242089 | PP837144 | *Nocardia salmonicida* DSM 4490^T^ | 99.65 |
|  | S3 | R_2_A | QTP243063 | PP837117 | *Arthrobacter yangruifuii* 785^T^ | 99.02 |
|  |  | R_2_A | QTP243071 | PP837126 | *Nocardia salmonicida* DSM 4490^T^ | 99.50 |
|  |  | R_2_A | QTP243079 | PP837134 | *Cellulomonas xylanilytica* XIL11^T^ | 98.59 |
|  |  | glucose-tryptone | QTP243090 | PP837145 | *Nocardia fluminea* DSM 44489^T^ | 99.50 |
|  |  | glucose-tryptone | QTP243097 | PP837152 | *Micromonospora aurantiaca* ATCC 27029^T^ | 99.72 |
|  |  | glucose-tryptone | QTP243121 | PP837183 | *Micromonospora saelicesensis* Lupac 09^T^ | 99.22 |
|  |  | glucose-tryptone | QTP243122 | PP837182 | *Micromonospora ureilytica* GUI23^T^ | 98.87 |
|  |  | glucose-tryptone | QTP243138 | PP837200 | *Streptosporangium amethystogenes* FYU S-5^T^ | 99.29 |
|  |  | glucose-tryptone | QTP243030 | PP837085 | *Nocardia fluminea* DSM 44489^T^ | 99.22 |
|  |  | glucose-tryptone | QTP243034 | PP837088 | *Agromyces terreus* DS-10^T^ | 99.30 |

*CRpfA represents the concentration of RpfA protein.

- **Supplementary Table 10.** Similarities of 16S rRNA genes between the non-*Streptomyces* strains of *Actinomycetota* isolated from different samples and their most similar type strains (Continue)

| CRpf(%)^*^ | Samples | Culture media | Strain numbers | Accession numbers | Most similar strains | Similarities (%) |
| --- | --- | --- | --- | --- | --- | --- |
| 10 | S3 | glucose-tryptone | QTP243035 | PP837089 | *Microbacterium* *phyllosphaerae* P 369/06^T^ | 99.65 |
|  |  | glucose-tryptone | QTP243139 | PP837202 | *Micromonospora* *pisi* GUI 15^T^ | 98.29 |
|  | S4 | R_2_A | QTP245004 | PP837059 | *Arthrobacter* *citreus* DSM 20133^T^ | 99.16 |
|  |  | R_2_A | QTP245129 | PP837212 | *Nocardia* *salmonicida* DSM 4490^T^ | 99.65 |
|  |  | glucose-tryptone | QTP245050 | PP837105 | *Oerskovia* *enterophila* HZD-2^T^ | 99.43 |
|  |  | glucose-tryptone | QTP245098 | PP837153 | *Oerskovia* *turbata* NBRC 15015^T^ | 99.79 |
|  |  | glucose-tryptone | QTP245099 | PP837154 | *Nocardia* *fluminea* DSM 44489^T^ | 99.15 |
|  | S5 | glucose-tryptone | QTP244022 | PP837077 | *Nocardia* *takedensis* DSM 44801^T^ | 99.36 |
|  |  | glucose-tryptone | QTP244134 | PP837220 | *Actinocorallia aurantiaca* JCM 8201^T^ | 99.43 |
|  | S6 | R_2_A | QTP246041 | PP837096 | *Embleya hyaline* NBRC 13850^T^ | 98.46 |
|  |  | R_2_A | QTP246042 | PP837097 | *Cellulomonas gelida* DSM 20111^T^ | 99.72 |
|  |  | R_2_A | QTP246045 | PP837100 | *Cellulomonas xylanilytica* XIL11^T^ | 98.94 |
|  |  | R_2_A | QTP246046 | PP837101 | *Cellulomonas timonensis* SN7^T^ | 99.08 |
|  |  | R_2_A | QTP246115 | PP837191 | *Rhodococcus coprophilus* DSM 43347^T^ | 99.71 |
|  |  | R_2_A | QTP246116 | PP837196 | *Micromonospora maoerensis* NEAU-MES19^T^ | 99.21 |
|  |  | R_2_A | QTP246117 | PP837197 | *Arthrobacter luteolus* CF-25^T^ | 98.66 |
|  |  | glucose-tryptone | QTP246051 | PP837106 | *Rhodococcus qingshengii* JCM 15477^T^ | 99.93 |
| 1 | S1 | R_2_A | QTP241167 | PP837255 | *Nocardia salmonicida* DSM 4490^T^ | 99.08 |
|  |  | R_2_A | QTP241169 | PP837257 | *Nocardia goodfellowii* A2012^T^ | 98.80 |
|  |  | R_2_A | QTP241153 | PP837241 | *Cellulomonas xylanilytica* XIL11^T^ | 98.66 |
|  |  | glucose-tryptone | QTP243147 | PP837236 | *Nocardia goodfellowii* A2012^T^ | 99.22 |

*CRpfA represents the concentration of RpfA protein.

- **Supplementary Table 10.** Similarities of 16S rRNA genes between the non-*Streptomyces* strains of *Actinomycetota* isolated from different samples and their most similar type strains (Continue)

| CRpf(%)^*^ | Samples | Culture media | Strain numbers | Accession numbers | Most similar strains | Similarities (%) |
| --- | --- | --- | --- | --- | --- | --- |
| 1 | S1 | glucose-tryptone | QTP241148 | PP837094 | *Nocardia* *salmonicida* DSM 4490^T^ | 99.50 |
|  |  | glucose-tryptone | QTP241149 | PP837237 | *Glycomyces* *algeriensis* NRRL B-16327^T^ | 99.72 |
|  | S2 | R_2_A | QTP242143 | PP837232 | *Nocardia* *fluminea* DSM 44489^T^ | 99.36 |
|  |  | R_2_A | QTP242150 | PP837238 | *Nocardia* *fluminea* DSM 44489^T^ | 99.43 |
|  |  | R_2_A | QTP242152 | PP837240 | *Oerskovia* *turbata* 27^T^ | 99.51 |
|  |  | R_2_A | QTP242144 | PP837233 | *Nocardia* *sungurluensis* CR3272^T^ | 98.94 |
|  |  | R_2_A | QTP242193 | PP837323 | *Embleya* *scabrispora* DSM 41855^T^ | 98.74 |
|  |  | R_2_A | QTP242171 | PP837259 | *Nocardia* *salmonicida* DSM 4490^T^ | 99.36 |
|  |  | glucose-tryptone | QTP242173 | PP837261 | *Nocardia* *salmonicida* R89^T^ | 99.50 |
|  |  | glucose-tryptone | QTP242166 | PP837254 | *Nocardia* *salmonicida* DSM 4490^T^ | 99.65 |
|  |  | glucose-tryptone | QTP242188 | PP837263 | *Nocardia* *grenadensis* NBRC 108939^T^ | 99.50 |
|  | S3 | R_2_A | QTP243161 | PP837249 | *Oerskovia* *turbata* 27^T^ | 99.79 |
|  |  | glucose-tryptone | QTP243182 | PP837186 | *Streptosporangium amethystogenes* FYU S-5^T^ | 99.15 |
|  |  | glucose-tryptone | QTP243165 | PP837253 | *Nocardia fluminea strain* DSM 44489^T^ | 99.43 |
|  |  | glucose-tryptone | QTP243184 | PP837207 | *Micromonospora profundi* DS3010^T^ | 99.51 |
|  | S4 | glucose-tryptone | QTP245164 | PP837252 | *Nocardia globerula* DSM 44596^T^ | 98.94 |
|  | S5 | R_2_A | QTP244185 | PP837188 | *Plantactinospora soyae* NEAU-gxj3^T^ | 98.79 |
|  |  | glucose-tryptone | QTP244191 | PP837266 | *Mumia zhuanghuii* Z350^T^ | 98.79 |
|  |  | glucose-tryptone | QTP244195 | PP837201 | *Nocardia takedensis* DSM 44801^T^ | 99.28 |
|  |  | glucose-tryptone | QTP244145 | PP837234 | *Oerskovia paurometabola* DSM 14281^T^ | 99.51 |
|  | S6 | R_2_A | QTP246156 | PP837244 | *Cellulomonas timonensis* SN7^T^ | 99.01 |
|  |  | R_2_A | QTP246170 | PP837258 | *Embleya hyaline* NBRC 13850^T^ | 98.26 |

*CRpfA represents the concentration of RpfA protein.

- **Supplementary Table 10.** Similarities of 16S rRNA genes between the non-*Streptomyces* strains of *Actinomycetota* isolated from different samples and their most similar type strains (Continue)

| CRpf(%)^*^ | Samples | Culture media | Strain numbers | Accession numbers | Most similar strains | Similarities (%) |
| --- | --- | --- | --- | --- | --- | --- |
| 1 | S6 | R_2_A | QTP246178 | PP837217 | *Micromonospora profundi* DS3010^T^ | 99.79 |
|  |  | R_2_A | QTP246194 | PP837267 | *Microlunatus aurantiacus* YIM 45721^T^ | 98.87 |
|  |  | glucose-tryptone | QTP246179 | PP837227 | *Mumia zhuanghuii* Z350^T^ | 98.87 |
| 0.1 | S1 | R_2_A | QTP241196 | PP837268 | *Cellulomonas xylanilytica* XIL11^T^ | 98.59 |
|  |  | glucose-tryptone | QTP241200 | PP837175 | *Nocardia goodfellowii* A2012^T^ | 99.42 |
|  | S2 | R_2_A | QTP242239 | PP837170 | *Nocardia salmonicida* DSM 4490^T^ | 99.65 |
|  |  | R_2_A | QTP242240 | PP837292 | *Nonomuraea aurantiaca* NEAU-L178^T^ | 98.53 |
|  |  | R_2_A | QTP242201 | PP837314 | *Oerskovia enterophila* DSM 43852^T^ | 98.87 |
|  |  | R_2_A | QTP242222 | PP837286 | *Nocardia salmonicida* DSM 4490^T^ | 99.72 |
|  |  | glucose-tryptone | QTP242199 | PP837271 | *Nocardia salmonicida* R89^T^ | 99.64 |
|  |  | glucose-tryptone | QTP242211 | PP837278 | *Isoptericola variabilis* MX5^T^ | 99.15 |
|  | S3 | R_2_A | QTP243206 | PP837273 | *Nocardia fluminea* DSM 44489^T^ | 99.36 |
|  |  | R_2_A | QTP243227 | PP837179 | *Micromonospora saelicesensis* Lupac 09^T^ | 99.43 |
|  |  | glucose-tryptone | QTP243225 | PP837289 | *Nocardia fluminea* DSM 44489^T^ | 99.36 |
|  |  | glucose-tryptone | QTP243228 | PP837190 | *Micromonospora saelicesensis* Lupac 09^T^ | 99.57 |
|  |  | glucose-tryptone | QTP243218 | PP837215 | *Rhodococcus coprophilus* DSM 43347^T^ | 99.71 |
|  |  | glucose-tryptone | QTP243220 | PP837285 | *Nocardia speluncae* N2 11^T^ | 99.22 |
|  |  | glucose-tryptone | QTP243210 | PP837277 | *Nocardia fluminea* DSM 44489^T^ | 99.43 |
|  | S5 | glucose-tryptone | QTP244234 | PP837290 | *Oerskovia turbata* 27^T^ | 99.79 |
|  |  | glucose-tryptone | QTP244238 | PP837216 | *Rhodococcus coprophilus* DSM 43347^T^ | 99.71 |
|  |  | glucose-tryptone | QTP244221 | PP837155 | *Tsukamurella tyrosinosolvens* DSM 44234^T^ | 99.86 |
|  |  | glucose-tryptone | QTP244197 | PP837269 | *Nocardia takedensis* DSM 44801^T^ | 99.29 |

*CRpfA represents the concentration of RpfA protein.

- **Supplementary Table 10.** Similarities of 16S rRNA genes between the non-*Streptomyces* strains of *Actinomycetota* isolated from different samples and their most similar type strains (Continue)

| CRpf(%)^*^ | Samples | Culture media | Strain numbers | Accession numbers | Most similar strains | Similarities (%) |
| --- | --- | --- | --- | --- | --- | --- |
| 0.1 | S6 | glucose-tryptone | QTP246226 | PP837184 | *Rhodococcus coprophilus* DSM 43347^T^ | 99.64 |
| 0 | S1 | R_2_A | QTP241254 | PP837303 | *Nocardia goodfellowii* A2012^T^ | 99.22 |
|  |  | R_2_A | QTP241255 | PP837304 | *Nocardia salmonicida* DSM 4490^T^ | 99.22 |
|  |  | R_2_A | QTP241245 | PP837295 | *Cellulomonas xylanilytica* XIL11^T^ | 98.87 |
|  |  | R_2_A | QTP241247 | PP837296 | *Cellulomonas humilata* ATCC 25174^T^ | 99.29 |
|  |  | R_2_A | QTP241264 | PP837312 | *Micromonospora auratinigra* TT1-11^T^ | 98.88 |
|  |  | glucose-tryptone | QTP241250 | PP837299 | *Williamsia limnetica* L1505^T^ | 98.46 |
|  |  | glucose-tryptone | QTP241241 | PP837317 | *Nocardia goodfellowii* A2012^T^ | 99.36 |
|  |  | glucose-tryptone | QTP241242 | PP837318 | *Nocardia salmonicida* DSM 4490^T^ | 99.36 |
|  | S2 | R_2_A | QTP242257 | PP837306 | *Nocardia salmonicida* R89^T^ | 99.72 |
|  |  | R_2_A | QTP242258 | PP837307 | *Nocardia fluminea* DSM 44489^T^ | 99.29 |
|  |  | R_2_A | QTP242244 | PP837294 | *Nocardia brasiliensis* DSM 43758^T^ | 98.45 |
|  |  | glucose-tryptone | QTP242253 | PP837302 | *Nocardia salmonicida* R89^T^ | 99.58 |
|  | S3 | glucose-tryptone | QTP243259 | PP837308 | *Mumia zhuanghuii* Z350^T^ | 99.44 |
|  |  | glucose-tryptone | QTP243260 | PP837309 | *Streptosporangium amethystogenes* FYU S-5^T^ | 99.43 |
|  |  | glucose-tryptone | QTP243261 | PP837310 | *Micromonospora zeae* NEAU-gq9^T^ | 99.72 |
|  |  | glucose-tryptone | QTP243262 | PP837311 | *Streptosporangium shengliense* NEAU-GH7^T^ | 99.37 |
|  |  | glucose-tryptone | QTP243263 | PP837199 | *Micromonospora saelicesensis* Lupac 09^T^ | 99.34 |
|  | S5 | glucose-tryptone | QTP245243 | PP837293 | *Nocardia takedensis* DSM 44801^T^ | 99.14 |
|  | S6 | R_2_A | QTP246256 | PP837305 | *Embleya hyaline* NBRC 13850^T^ | 98.32 |

*CRpfA represents the concentration of RpfA protein.

- **Supplementary** **Table 11.** Quantities and proportions of different genera of *Actinomyceota* isolated by adding different the concentrations of RpfA protein

| Genera | Numbers(strain) and proportion (%) | | | |  |
| --- | --- | --- | --- | --- | --- |
|  | 0% CRpfA | 0.1% CRpfA | 1% CRpfA | 10% CRpfA | |
| *Streptomyces* | 5 (20.83%) | 25 (55.56%) | 27 (48.21%) | 80 (57.55%) | |
| *Nocardia* | 9 (37.50%) | 9 (20.00%) | 14 (25.00%) | 19 (13.67%) | |
| *Cellulomonas* | 2 (8.33%) | 1 (2.22%) | 2 (3.57%) | 9 (6.47%) | |
| *Micromonospora* | 3(12.50%) | 2 (4.44%) | 2 (3.57%) | 9 (6.47%) | |
| *Oerskovia* | — | 2 (4.44%) | 3 (3.57%) | 6 (4.32%) | |
| *Isoptericola* | — | 1 (2.22%) | — | 3 (2.16%) | |
| *Arthrobacter* | — | — | — | 3 (2.16%) | |
| *Rhodococcus* | — | 3(6.67%) | — | 2 (1.44%) | |
| *Embleya* | 1 (4.17%) | — | 2 (3.57%) | 2 (1.44%) | |
| *Nonomuraea* | — | 1 (2.22%) | — | 1 (0.72%) | |
| *Streptosporangium* | 2 (8.33%) | — | 1 (1.79%) | 1 (0.72%) | |
| *Actinocorallia* | — | — | — | 1 (0.72%) | |
| *Agromyces* | — | — | — | 1 (0.72%) | |
| *Microbacterium* | — | — | — | 1(0.72%) | |
| *Promicromonospora* | — | — | — | 1 (0.72%) | |
| *Mumia* | 1 (4.17%) | — | 2 (3.57%) | — | |
| *Glycomyces* | — | — | 1 (1.79%) | — | |
| *Microlunatus* | — | — | 1 (1.79%) | — | |
| *Tsukamurella* | — | 1 (2.22%) | — | — | |
| *Williamsia* | 1 (4.17%) | — | — | — | |
| *Plantactinospora* | — | — | 1 (1.79%) | — | |
| Total | 24 | 45 | 56 | 139 | |

CRpfA represents the concentration of RpfA protein.

- **Supplementary** **Table 12.** The potential new species strains of phylum *Actinomycetota* and the similarities between them and the most similar type strains

| Samples | Strain numbers | Accession numbers | CRpfA (%) | Culture media | Most similar strains | Similarities (%) |
| --- | --- | --- | --- | --- | --- | --- |
| S1 | QTP241024 | PP837079 | 10 | R_2_A | *Cellulomonas xylanilytica* XIL11^T^ | 98.52 |
|  | QTP241057 | PP837111 | 10 | Glucose-tryptone | *S．tateyamensis* Sp080513SC-30^T^ | 97.82 |
|  | QTP241175 | PP837313 | 1.0 | R_2_A | *S．tateyamensis* Sp080513SC-30^T^ | 98.07 |
|  | QTP241196 | PP837268 | 0.1 | R_2_A | *Cellulomonas xylanilytica* XIL11^T^ | 98.59 |
|  | QTP241248 | PP837297 | 0 | Glucose-tryptone | *S. tateyamensis* Sp080513SC-30^T^ | 97.82 |
|  | QTP241250 | PP837299 | 0 | Glucose-tryptone | *Williamsia limnetica* L1505^T^ | 98.46 |
| S2 | QTP242018 | PP837073 | 10 | R_2_A | *S．chlorus* BK125^T^ | 98.52 |
|  | QTP242244 | PP837294 | 0 | R_2_A | *Nocardia brasiliensis* DSM 43758^T^ | 98.45 |
|  | QTP242055 | PP837109 | 10 | R_2_A | *S．cahuitamycinicus* 13K301^T^ | 98.24 |
|  | QTP242240 | PP837292 | 0.1 | R_2_A | *Nonomuraea aurantiaca* NEAU-L178^T^ | 98.53 |
|  | QTP242095 | PP837150 | 10 | R_2_A | *S. cahuitamycinicus* 13K301^T^ | 98.39 |
|  | QTP242107 | PP837161 | 10 | R_2_A | *S. cahuitamycinicus* 13K301^T^ | 98.25 |
|  | QTP242111 | PP837164 | 10 | R_2_A | *S. cahuitamycinicus* 13K301^T^ | 98.38 |
| S3 | QTP243139 | PP837202 | 10 | Glucose-tryptone | *Micromonospora pisi* GUI 15^T^ | 98.29 |
|  | QTP243079 | PP837134 | 10 | R_2_A | *Cellulomonas xylanilytica* XIL11^T^ | 98.59 |
| S4 | QTP245026 | PP837081 | 10 | R_2_A | *S. lacrimifluminis* Z1027^T^ | 98.31 |
|  | QTP245198 | PP837270 | 0.1 | R_2_A | *S. lacrimifluminis* Z1027^T^ | 98.53 |
| S6 | QTP246041 | PP837096 | 10 | R_2_A | *Embleya hyaline* NBRC 13850^T^ | 98.46 |
|  | QTP246170 | PP837258 | 1.0 | R_2_A | *Embleya hyaline* NBRC 13850^T^ | 98.26 |
|  | QTP246177 | PP837181 | 1.0 | R_2_A | *S．urticae* NEAU-PCY-1^T^ | 98.32 |
|  | QTP246256 | PP837305 | 0 | R_2_A | *Embleya hyaline* NBRC 13850^T^ | 98.32 |

CRpfA represents the concentration of RpfA protein.

***Streptomyces lusitanus* HBU208066^T^**

*Streptomyces thermocarboxydus* NBRC 16323^T^

*Streptomyces hydrogenans* NBRC 13475^T^

***Streptomyces coelicolor* CGMCC 4.1658^T^**

***Streptomyces niveus* JCM 4251^T^**

***Streptomyces laurentii* JCM 5063^T^**

*Streptomyces termitum* NBRC 13087^T^

***Streptomyces griseus* CGMCC 4.1419^T^**

*Streptomyces anulatus* NBRC 13369^T^

0.0050

- **Supplementary Figure 1.** Phylogenetic tree constructed based on 60 type strains of *Streptomyces* species


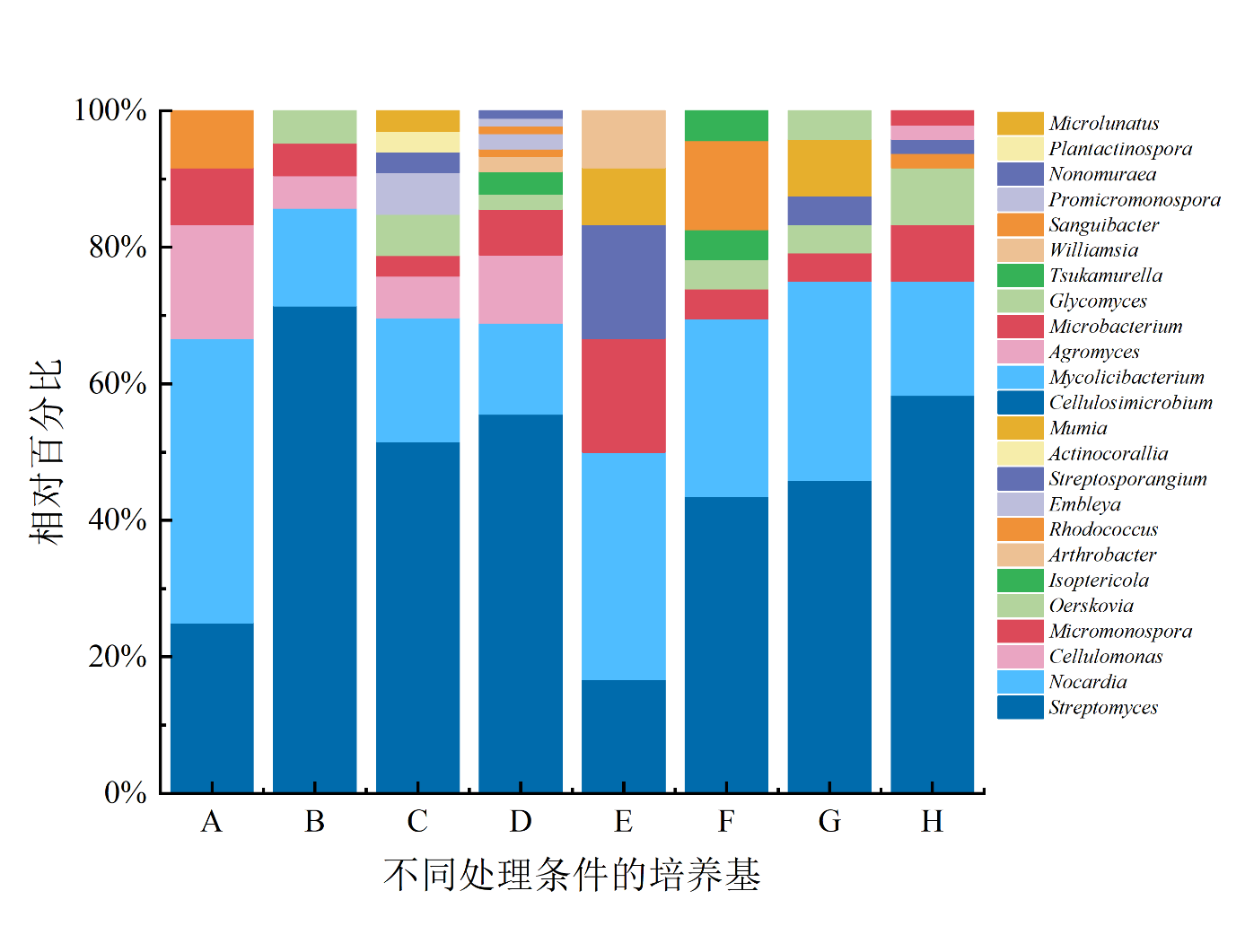


- **Supplementary** **Figure 2.** Statistical map of the diversity of *Actinomycetota* members isolated from six samples at the generic level. (A) R_2_A+CRpf 0%; (B) R_2_A+CRpf 0.1%; (C) R_2_A+CRpf 1%; (D) R_2_A+CRpf 10%; (E) GT + CRpf 0%; (F) GT + CRpf 0.1%; (G) GT + CRpf 1%; (H) GT + CRpf 10%; GT represents Glucose-tryptone, CRpfA represents the concentration of RpfA protein.
